# Experience-dependent changes in hippocampal spatial activity and hippocampal circuit function are disrupted in a rat model of Fragile X Syndrome

**DOI:** 10.1186/s13229-022-00528-z

**Published:** 2022-12-20

**Authors:** Antonis Asiminas, Sam A. Booker, Owen R. Dando, Zrinko Kozic, Daisy Arkell, Felicity H. Inkpen, Anna Sumera, Irem Akyel, Peter C. Kind, Emma R. Wood

**Affiliations:** 1 Centre for Discovery Brain Sciences, University of Edinburgh, Edinburgh, EH8 9XD UK; 2 Simons Initiative for the Developing Brain, University of Edinburgh, Edinburgh, EH8 9XD UK; 3 Patrick Wild Centre, University of Edinburgh, Edinburgh, EH8 9XD UK; 4 UK Dementia Research Institute at the Edinburgh Medical School, University of Edinburgh, Edinburgh, EH8 9XD UK; 5 Centre for Brain Development and Repair, Bangalore, 560065 India; 6 Present Address: Center for Translational Neuromedicine, Faculty of Health and Medical Sciences, University of Copenhagen, 2200 Copenhagen, Denmark

## Abstract

**Background:**

Fragile X syndrome (FXS) is a common single gene cause of intellectual disability and autism spectrum disorder. Cognitive inflexibility is one of the hallmarks of FXS with affected individuals showing extreme difficulty adapting to novel or complex situations. To explore the neural correlates of this cognitive inflexibility, we used a rat model of FXS (*Fmr1*^−/*y*^).

**Methods:**

We recorded from the CA1 in *Fmr1*^−/*y*^ and WT littermates over six 10-min exploration sessions in a novel environment—three sessions per day (ITI 10 min). Our recordings yielded 288 and 246 putative pyramidal cells from 7 WT and 7 *Fmr1*^−/*y*^ rats, respectively.

**Results:**

On the first day of exploration of a novel environment, the firing rate and spatial tuning of CA1 pyramidal neurons was similar between wild-type (WT) and *Fmr1*^−/*y*^ rats. However, while CA1 pyramidal neurons from WT rats showed experience-dependent changes in firing and spatial tuning between the first and second day of exposure to the environment, these changes were decreased or absent in CA1 neurons of *Fmr1*^−/*y*^ rats. These findings were consistent with increased excitability of *Fmr1*^−/*y*^ CA1 neurons in ex vivo hippocampal slices, which correlated with reduced synaptic inputs from the medial entorhinal cortex. Lastly, activity patterns of CA1 pyramidal neurons were dis-coordinated with respect to hippocampal oscillatory activity in *Fmr1*^−/*y*^ rats.

**Limitations:**

It is still unclear how the observed circuit function abnormalities give rise to behavioural deficits in *Fmr1*^−/*y*^ rats. Future experiments will focus on this connection as well as the contribution of other neuronal cell types in the hippocampal circuit pathophysiology associated with the loss of FMRP. It would also be interesting to see if hippocampal circuit deficits converge with those seen in other rodent models of intellectual disability.

**Conclusions:**

In conclusion, we found that hippocampal place cells from *Fmr1*^−/*y*^ rats show similar spatial firing properties as those from WT rats but do not show the same experience-dependent increase in spatial specificity or the experience-dependent changes in network coordination. Our findings offer support to a network-level origin of cognitive deficits in FXS.

**Supplementary Information:**

The online version contains supplementary material available at 10.1186/s13229-022-00528-z.

## Background

Fragile X syndrome (FXS) is a genetic neurodevelopmental disorder caused by a CGG repeat expansion in the promoter region of the *FMR1* gene, resulting in its epigenetic silencing and subsequent loss of its protein product FMRP [1–3]. Although FXS is a rare disorder, it is one of the most common inherited forms of intellectual disability (ID) and autism spectrum disorder (ASD) [4, 5]. FMRP has numerous functions, from translational repression of a wide variety of mRNAs to directly binding ion channels to regulate excitability [6–8]. Hence, elucidating direct mechanistic links between FMRP loss and specific behavioural phenotypes has been challenging. Bridging this gap requires an understanding of the functional changes to neural circuits underlying behaviour resulting from the absence of FMRP.

Rodent models of FXS have been instrumental in understanding how FMRP loss leads to behavioural and cognitive changes. *Fmr1*^−/*y*^ mice exhibit deficits in a range of cognitive tasks, as well as synaptic plasticity abnormalities in various brain areas [9, 10]. Hippocampal circuits are affected in both FXS patients and rodent models of FXS, and hippocampal pathophysiology has been well characterized in rodent models [11]. Studies in *Fmr1*^−/*y*^ mice have identified changes in CA1 pyramidal neuron excitability [12–14] and the strength of inputs they receive [12], alterations in hippocampal synaptic plasticity [11, 15, 16] and deficits in some hippocampus-dependent learning and memory tasks [11, 17]. Consistent with these findings, we have reported deficits in a subset of hippocampus-dependent memory tasks and disturbances in hippocampal plasticity in *Fmr1*^−/*y*^ rat models of FXS [18, 19].

Recent studies have begun to shed light on the effects of FMRP loss on hippocampal function, using in vivo electrophysiological recordings from the hippocampus of freely moving mice. The specific analyses and range of findings are variable. However, with the exception of one study [20], slow gamma oscillations (20–55 Hz) within the pyramidal cells layer in CA1 appear to be stronger in *Fmr1*^−/*y*^ mice compared to WT littermates [21–23]. Moreover, the organization of pyramidal activity by theta and gamma oscillations is shown to be abnormal in *Fmr1*^−/*y*^ mice, with pyramidal cells firing preferentially in later theta and earlier gamma (40 Hz) phases, while pyramidal cell population activity is less complex than that from WT littermates [20]. Studies investigating the effect of FMRP loss on CA1 pyramidal cell activity at the single neuron level have produced more variable findings. Consistent with the excitability changes reported in vitro [12–14] Boone et al. [21] reported that CA1 pyramidal neurons in *Fmr1*^−/*y*^ mice were hyperactive (increased mean firing rate) during both sleep and wake states (including during movement). In contrast, two other studies have reported no differences in the mean firing rates of WT and *Fmr1*^−/*y*^ CA1 pyramidal neurons during movement [20, 24].

A well-characterized feature of hippocampal pyramidal neurons is their spatially modulated firing, such that a given neuron fires when the animal moves through a specific region of the environment (the cell’s place field; [25]). The activity of hippocampal place cells is crucial both for spatial navigation, and for the encoding and consolidation of new spatial and episodic memories [26]. Previous studies investigating the spatial firing properties of CA1 place cells in *Fmr1*^−/*y*^ mice have also yielded discrepant findings, with Talbot et al. [20] reporting no differences from WT mice across a range of spatial measures including spatial information, spatial coherence, and proportion of the environment in which the cell was active, whereas Arbab et al. [24] reported impaired spatial coding in *Fmr1*^−/*y*^ mice, indicated by place cells firing across a larger proportion of the environment, with larger place fields, and reduced spatial specificity of firing. Arbab et al. also reported reduced short-term stability of the firing rate maps (i.e. where in the environment the cell fired) in *Fmr1*^−/*y*^ mice.

The encoding of environments by hippocampal place cells is a fundamental learning process that is supported by cellular mechanisms involved in long-term plasticity [27]. CA1 pyramidal neurons form place fields during an animal’s first exposure to a novel environment, but the spatial tuning and stability of their fields improve as a function of experience, and these changes are dependent on different plasticity processes within the hippocampal formation [11, 28–33]. Thus, the familiarity of the environment may influence whether differences are observed between WT and *Fmr1*^−/*y*^ place cells, both in firing rate and spatial firing properties. The recording protocols used in the mouse studies described above [20, 21, 24] varied in several ways, including the familiarity/novelty of the recording environments used, which may have contributed to the disparate findings.

The primary aim of the current study was to examine hippocampal CA1 pyramidal neuron activity, spatial coding, and temporal firing in the Long Evans *Fmr1*^−/*y*^ rat model. We recorded from the CA1 pyramidal neuron layer of the dorsal hippocampus in *Fmr1*^−/*y*^ and WT littermate rats as they explored an initially novel environment over three 10-min exploration sessions on each of two consecutive days, which allowed us to examine experience-dependent features of place cell activity and spatial coding as rats became familiar with an initially novel environment. This, in turn, allowed us to address whether some of the disparate findings reported in *Fmr1*^−/*y*^ mice may be related to the animal’s experience in the environment. To determine the cellular mechanisms of any alterations, we also performed ex vivo slice recordings from CA1 pyramidal neurons to examine their intrinsic and synaptic properties.

Based on our previous observations that *Fmr1*^−/*y*^ rats exhibit hippocampal plasticity abnormalities and deficits in hippocampus-dependent recognition memory [18, 19], and the reduced cognitive flexibility in individuals with FXS [34], we predicted that experience-dependent changes in the spatial tuning and the spatial stability of hippocampal representations in novel environments would be disrupted in *Fmr1*^−/*y*^ rats. Based on the findings in *Fmr1*^−/*y*^ mice, we also predicted that gamma oscillations will be stronger in *Fmr1*^−/*y*^ rats and that pyramidal cell spiking patterns will be discoordinated in relation to theta and gamma oscillations.

## Methods

### Animals

Subjects were adult male Long-Evans Hooded WT and *Fmr1*^em1/PWC^ rats, hereafter referred to as *Fmr1*^−/*y*^ (for more details on this rat model see [19]). The rats were bred in-house and kept on a 12 h/12 h light/dark cycle with ad libitum access to food and water unless noted below. Following weaning (postnatal day 21) up until the start of the experiment, *Fmr1*^−/*y*^ and WT littermates were group-housed (3 to 5 rats per cage) in mixed-genotype cages. Animals were selected pseudo-randomly from a litter for use in the experiment (cohorts of 2–6 animals at a time). Each cohort included both WT and *Fmr1*^−/*y*^ rats. Selection of animals was done by an experimenter not involved in any stage of the experiment, data collection or analysis, by randomly picking rat ID numbers from a given litter (while ensuring balance of WT and *Fmr1*^−/*y*^ rats). Experimenters involved in data collection and data analysis were blind to the genotype of the subjects throughout all stages of the experiments and data analysis until final statistical analyses were conducted [in line with the ARRIVE (Animal Research: Reporting of In Vivo Experiments) guidelines [35]. All animal experiments were approved by the University of Edinburgh veterinary services before their start. Procedures were performed in accordance with the guidelines established by European Community Council Directive 2010/63/EU (22 September 2010) and by the Animal Care (Scientific Procedures) Act 1986, and under the authority of Home Office Licences.

For ex vivo experiments, subjects were 14 WT and 14 *Fmr1*^−/*y*^ rats aged 2–3 months. For in vivo experiments, subjects were 7 WT and 7 *Fmr1*^−/*y*^ adult rats aged 3–4 months at the time of surgery. After surgery, these rats were housed individually in cages designed to minimize head-stage damage, and after recovery from surgery, they were food restricted such that they maintained approximately 90% of their free-feeding weight. All in vivo recordings were conducted during the light phase of the cycle.

### In vivo experiments: electrodes and surgery

The microdrives used for the in vivo recordings were based on a modified tripod design described previously [36]. The drives were loaded with eight tetrodes, each of which was composed of four HML coated, 17 µm, 90% platinum 10% iridium wires (California Fine Wire, Grover Beach, CA). Tetrodes were threaded through a thin-walled stainless steel cannula (23 Gauge Hypodermic Tube, Small Parts Inc, Miramar, FL). The tip of every wire was gold-plated (Non-Cyanide Gold Plating Solution, Neuralynx, MT) to reduce the impedance of the electrode from a resting impedance of 0.7–0.9 MΩ to a plated impedance in the range of 150–250 kΩ (200 kΩ being the target impedance) one to ten hours before surgery. Electrodes were implanted using standard stereotaxic procedures under isoflurane anaesthesia. Hydration was maintained by subcutaneous administration of 2.5 ml 5% glucose and 1 mL 0.9% saline. Animals were also given an anti-inflammatory analgesia (small animal Carprofen/Rimadyl, Pfizer Ltd., UK) subcutaneously. Electrodes were lowered to just above the dorsal CA1 cell layer of the hippocampus (-3.5 mm AP from bregma, + 2.4 mm ML from the midline, − 1.7 mm DV from dura surface). The drive assembly was anchored to the skull screws and bone surface using dental acrylic (Associated Dental Products Ltd. Swindon, UK). Animals were monitored closely for at least two hours in their home cage while recovering from anaesthesia, and then returned to the colony. Following this, at least one week of recovery time passed before access to food was restricted and screening for cellular activity began.

### In vivo unit recording

In vivo single unit and local field potential (LFP) activity was recorded using a 32-channel Axona USB system (Axona Ltd., St. Albans, UK). Mill-Max connectors built into the rat’s microdrive were attached to the recording system via two unity gain buffer amplifiers and a light, flexible, elasticated recording cable. The recording cable passed signals through a ceiling mounted slip-ring commutator (Dragonfly Research and Development Inc., Ridgeley, West Virginia) to a pre-amplifier where they were amplified 1000 times. The signal was then passed to a system unit; for single unit recording the signal was band-pass (Butterworth) filtered between 300 and 7000 Hz. Signals were digitized at 48 kHz (50 samples per spike, 8 bits/sample) and could be further amplified 10–40 times at the experimenter’s discretion. The LFP signals were recorded from one channel of a tetrode located in the pyramidal neuron layer of the dCA1 area. The signals were amplified by a factor of 1000–2000, low-pass filtered at 500 Hz, and sampled at a rate of 4.8 kHz (16 bits/sample). A notch filter was applied at 50 Hz. The position of the animal was recorded by tracking two small light-emitting diodes fixed on the headstage connected to the rat’s microdrive. A ceiling-mounted, infrared sensitive CCTV camera tracked the animal’s position at a sampling rate of 50 Hz. Rats were screened for single unit activity and for the presence of theta oscillations once or twice a day, at least five days a week, while foraging for chocolate treats (CocoPops, Kellogg's, Warrington, UK). The screening environment was a blue, wooden square arena (1 m × 1 m × 52 cm) which was not used during later experiments. At the end of each screening session, rats were removed from the recording apparatus and the electrodes were lowered if no hippocampal unit activity had been observed. Brain tissue was allowed to recover from electrode movement for at least 5 h before a new screening session started.

### In vivo recording protocol

**Fig. 1:**
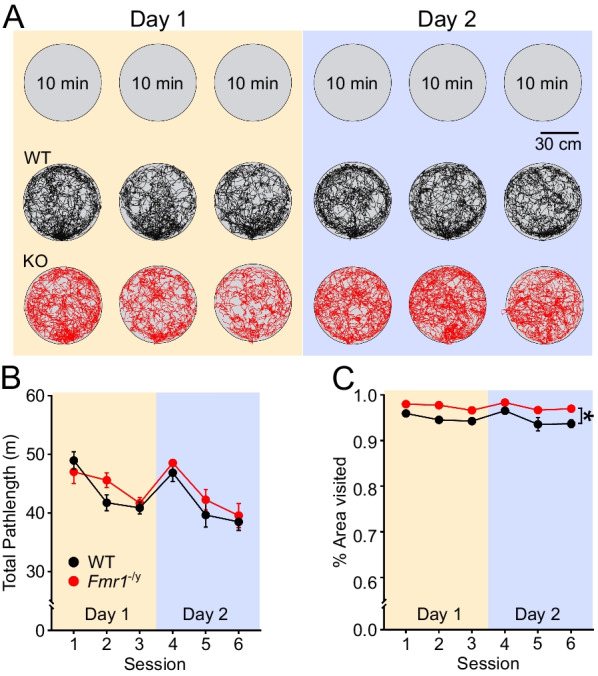
WT and *Fmr1*^−/*y*^ rats move similar distances and at similar speeds while foraging in a novel environment over the two days, but *Fmr1*^−/*y*^ rats visit more of the environment than WT rats. **A** Schematic of the recording protocol (Top) and example trajectories from a WT (black) and an *Fmr1*^−/*y*^ rat (red). **B** Total path length decreased across the three sessions within a day for both WT and *Fmr1*^−/*y*^ rats, but there was no difference between genotypes or across days (session-in-day *p* < 0.0001; day *p* = 0.069; genotype *p* = 0.967). **C** Both WT and *Fmr1*^−/*y*^ rats showed good coverage of the environment in all recording sessions. *Fmr1*^−/*y*^ rats visited a significantly higher proportion of the environment than WT rats (*genotype *p* = 0.001). There was also a decrease in exploration across sessions within each day, but not across days (session-in day *p* = 0.001; day *p* = 0.615). Data points depict rat group means; error bars depict SEM; Statistical analyses used 3-way ANOVA with genotype (between subjects), day (within subjects) and session-in day (within subjects); *n*_WT_ = 7 rats, *n Fmr1*^−/*y*^ = 7 rats. Pale yellow and pale purple backgrounds denote data from Day 1 and Day 2, respectively

Once at least 10 putative CA1 pyramidal neurons were detected, the rat was transferred to a totally novel grey plastic cylindrical environment (62 cm diameter, 60 cm walls) surrounded by black curtains and different lighting within the same experimental room. The cylinder contained one salient black cue card that remained stable throughout the two days of recording. Three 10-min recording sessions, separated by a 10-min inter-session interval (ISI), took place on each of the two days of the experiment, during which the rat foraged for scattered chocolate cereal treats in the recording arena (six sessions in total) (Fig. 1A). Between sessions of the same day, that rat was placed in a plastic holding bucket (25 cm diameter, sawdust bedding) while remaining tethered.

### Single unit analysis

Single unit activity was analysed offline using a custom-written MATLAB (MathWorks) routine that makes use of the Klustakwik spike-sorting program [37]. Electrophysiological recordings from all six sessions of the experiment were combined before the use of the spike sorting algorithms. This permits tracking of clusters across sessions and days that does not rely on assumptions about stability of cluster boundaries. The dimensionality of the waveform information was reduced to the first principal component, energy, peak amplitude, peak time, and width of the waveform. The energy of a signal *x* was defined as the sum of squared moduli given by the formula:

$$\varepsilon_{x} \triangleq \mathop \sum \limits_{n = 0}^{N - 1} \left| {x_{n} } \right|^{2}$$

Based on these parameters, Klustakwik spike sorting algorithms were then used to distinguish and isolate separate clusters. The clusters were then further checked and refined manually using the manual cluster cutting GUI, Klusters [38]. In addition to the aforementioned waveform features, manual cluster cutting also made use of spike auto- and cross-correlograms to examine refractory period and complex spiking. Cluster quality was operationalized by calculating isolation distance (Iso-D), *L*_ratio_, and peak waveform amplitude, taken as the highest amplitude reached by the four mean cluster waveforms. For cluster *C*, containing *n*_*c*_ spikes, Iso-D is defined as the squared Mahalanobis distance of the *n*_*c*_*-th* closest *non-c* spike to the centre of *C*. The squared Mahalanobis distance was calculated as:

$$D_{i,C}^{2} = \left( {x_{i} - \mu_{C} } \right)^{T} \Sigma_{c}^{ - 1} \left( {x_{i} - \mu_{C} } \right)$$

where *x*_*i*_ is the vector containing features for spike *i*, and *μ*_*C*_ is the mean feature vector for cluster *C*, and *Σ*_*C*_ is the covariance matrix of the spikes in cluster *C*. A higher value indicates better isolation from non-cluster spikes [39]. The L quantity was defined as:

$$L_{\left( c \right)} = \mathop \sum \limits_{i \notin C} 1 - {\text{CDF}}_{{x_{df}^{2} }} \left( {D_{i,C}^{2} } \right)$$

where $i \notin C$ is the set of spikes which are not members of the cluster and CDF is the cumulative distribution function of the distribution with 8 degrees of freedom. The cluster quality measure, *L*_ratio_ was thus defined as *L* divided by the total number of spikes in the cluster [40]. Finally, cluster waveforms were visually inspected to ensure that waveforms of a given cluster looked similar across the 6 recording sessions.

A cluster was classified as a pyramidal neuron if it satisfied the following criteria: i) Iso-D > 15 and *L*_ratio_ < 0.2 and ii) the width of its average waveform was > 250 μs and mean firing rate was < 5 Hz. A pyramidal cell was considered to be active in a given session if the mean firing rate was greater than 0.1 Hz (but less than 5 Hz). Only spikes that occurred during periods of animal locomotion (speed > 3 cm/s) were included in the analyses.

To calculate burst probability, we first defined bursts as groups of spikes with interspike interval (ISI) < 10 ms. Using ISIs of 6, 9 and 12 ms yielded consistent results. The number of bursts *N*_*B*_ and single spikes *N*_*S*_ in each session were counted for each cell. The burst probability was calculated as *N*_*B*_*/*(*N*_*B*_ + *N*_*S*_) [41, 42]. A similar analysis of cell bursting behaviour using a different definition (numbers of spikes in bursts divided by total spikes) [43] yields similar results (data not shown).

Several measures of the spatial activity were calculated for each active pyramidal cell in a given session:

Spatial information content is given by the equation:

$${\text{Information}} \;{\text{content}} = \sum P_{i} \left( {R_{i} {/}R} \right)\log_{2} \left( {R_{i} {/}R} \right)$$

where *i* is the bin number, *Pi* is the probability for occupancy of bin *i*, *Ri* is the mean firing rate for bin *i*, and *R* is the overall average firing rate [44].

Sparsity was calculated as previously[45]:

$${\text{Sparsity}} = \left( {\sum P_{i} R_{i} } \right)^{2} /\sum P_{i} R_{i}^{2}$$

where *i* is the bin number, *Pi* is the probability for occupancy of bin *i* and *Ri* is the mean firing rate for bin *i*.

Firing rate maps were produced for each session by dividing the recording cylinder area into a grid of 2.5 cm square bins. The firing rate in each bin was calculated as the total number of spikes which occurred in that bin divided by the total length of time spent there. Bins in which the rats spent less than 100 ms were treated as if they had not been visited. These bin-specific firing rates were plotted in a heat map, showing where the preferred firing location of a cell was in a given environment. The rate maps were generated using an algorithm described by the following equations.

The Gaussian kernel used is given by:

$$g\left( x \right) = \exp \left( {\frac{{ - x^{2} }}{2}} \right)$$

The summary algorithm for calculating firing rate for each spatial bin is then given by:

$$\lambda \left( x \right) = \mathop \sum \limits_{i = 1}^{n} g\left( {\frac{{S_{i - x} }}{h}} \right){/}\mathop \smallint \limits_{0}^{T} g\left( {\frac{y\left( t \right) - x}{h}} \right){\text{d}}t$$

where *S*_*i*_ represents the positions of every recorded spike, *x* is the centre of the current bin, the period [0, *T*] is the recording session time period, *y(t)* is the position of the rat at time *t*, and *h* is a smoothing factor, which was set to 2.5 cm.

The percentage of active bins for each session was calculated by dividing the number of bins in the firing rate map which contained spikes by the number of bins visited. Place fields were defined as areas in the firing rate maps of at least 9 contiguous bins with firing rate > 20% of maximum bin firing rate [46]. In cases where secondary place fields were detected, only the main place field was included in the analysis of place field size.

To assess the stability of spatial firing, we calculated the Pearson correlation between firing rate maps of successive recording sessions. This analysis was only conducted on identified cells which exhibited spatial firing, defined as SI > 0.5 bits per spike, in both sessions.

### Spectral analysis

Position data from each session were binned into 500-ms epochs, and the velocity for each epoch was calculated. Raw LFP traces (4.8 kHz) were z-scored (mean was subtracted and divided by standard deviation). LFP data analysis was done on all the 4 s periods of activity (> 3 cm/s) following periods of immobility. Time–frequency spectrograms were calculated using Chronux Toolbox [http://chronux.org; [47]], function *mtspecgramc()* using a window size and time step of 20 s and 10 s, respectively [48]. Power estimates for the frequency bands of interest [Theta (6–12 Hz), Slow Gamma (30–45 Hz), Medium Gamma (55–100 Hz)] were excised from the spectrogram and averaged. The average spectrograms for each genotype group in each session are represented in Additional file 1: Fig. S9. For the relationships between running speed and oscillation power, the recording was divided into 500-ms bins and EEG signals from all 500-ms bins were stratified based on the velocity (3 cm/s wide bins). Power spectra estimation during each bin was done by means of the Welch periodogram method (50% overlapping Hamming windows), which was obtained by using the *pwelch()* function from MATLAB Signal Processing Toolbox. Specific band powers were computed by integrating the power spectral density (PSD) estimate for each frequency range of interest [MATLAB function *bandpower()*].

### Phase-locking analysis

To investigate spike timing with respect to oscillations, a band-pass filter was applied to the LFP signals. The low cut-off stop band was the low passband minus 2 Hz; the high cut-off stop band was the high passband plus 2 Hz [Theta (4–14 Hz), Slow Gamma (28–47 Hz), Medium Gamma (58–102 Hz)]. Both the instantaneous amplitude and the phase time series of a filtered signal were computed from the Hilbert transform, which was obtained by using the *hilbert()* function from the MATLAB Signal Processing Toolbox. Only spikes during bins (500 ms) of strong oscillations (> 2 standard deviations of mean power) were included in this analysis [49, 50]. Every recorded spike from these periods was assigned a spike phase *θ*_*j*_, where *j* denotes the *j-th* spike. The mean resultant vector r was calculated as:

$$r = \mathop \sum \limits_{j} \exp \left( {i\theta_{j} } \right){/}N$$

where *N* is the total number of spikes. The strength of phase locking (resultant length) was defined as $\left| r \right|$. Theoretically, this value ranges from 0 to 1. The value is zero if the phases are uniformly distributed along the phases of gamma oscillations, while it is one if all spikes fire at exactly the same phase. In practice, the values for individual neurons are distributed mostly in the range of 0–0.2 as shown in Fig. 7 and Additional file 1: Fig S11. The trough of gamma oscillation was defined as 0/360°.

### Histology

At the end of the in vivo recording experiments, animals were given an overdose of pentobarbital intraperitoneally (Euthatal, Merial Animal Health Ltd., Essex, UK) and perfused with 0.9% saline solution followed by a 4% formalin solution. The brain was extracted and stored in 4% formalin for at least seven days prior to any histological analyses. The brains were sliced in 32-µm sections on a freezing microtome at − 20°. These sections were stained with a 0.1% Cresyl violet solution and the tissue section that best revealed the electrode track was imaged using ImageJ software (ImageJ, NIH, Bethesda).

### Ex vivo electrophysiology

Ex vivo brain slices were prepared as previously described [12]. Briefly, rats were sedated with isoflurane, anaesthetized with sodium pentobarbital (100 mg/kg) and then transcardially perfused with ice-cold, carbogenated (95% O2/5% CO2), and filtered, sucrose-modified artificial cerebrospinal fluid (sucrose-ACSF; in mM: 87 NaCl, 2.5 KCl, 25 NaHCO_3_, 1.25 NaH_2_PO_4_, 25 glucose, 75 sucrose, 7 MgCl_2_, 0.5 CaCl_2_). Once perfused, the brain was rapidly removed and 400 μm slices containing the dorsal pole of the hippocampus were cut on an oscillating blade vibratome (VT1200S, Leica, Germany) in the coronal plane. Slices were then transferred to a submerged chamber containing sucrose-ACSF at 35 °C for 30 min, then at room temperature until needed.

For recording, slices were transferred to a submerged chamber perfused with pre-warmed carbogenated ACSF (in mM: 125 NaCl, 2.5 KCl, 25 NaHCO_3_, 1.25 NaH_2_PO_4_, 25 glucose, 1 MgCl_2_, 2 CaCl_2_) at a flow rate of 4–6 mL.min^-1^ at 31 ± 1 °C) which contained 50 µM picrotoxin to block GABA_A_ receptor-mediated currents. Neurons were visualized under infrared differential interference contrast (IR-DIC) microscopy with a digital camera (SciCamPro, Scientifica, UK) mounted on an upright microscope (SliceScope, Scientifica, UK) with a 40 × water-immersion objective lens (1.0 N.A., Olympus, Japan). Whole-cell patch-clamp recordings were performed with a Multiclamp 700B (Molecular Devices, CA, USA) amplifier, using recording pipettes pulled from borosilicate glass capillaries (1.5 mm outer/0.86 mm inner diameter, Harvard Apparatus, UK) on a horizontal electrode puller (P-97, Sutter Instruments, CA, USA). Pipettes were filled with a K-gluconate based internal solution (in mM 142 K-gluconate, 4 KCl, 0.5 EGTA, 10 HEPES, 2 MgCl_2_, 2 Na_2_ATP, 0.3 Na_2_GTP, 1 Na_2_Phosphocreatine, 2.7 Biocytin, pH = 7.4, 290–310 mOsm) which gave a 3–5 MΩ tip resistance. Neurons were rejected if: they were more depolarized than − 50 mV, had an access resistance > 30 MΩ, or the access resistance changed by more than 20% during the recording. Cell-attached recordings were performed as above, but without breaking through into the whole-cell configuration.

Intrinsic membrane properties were measured in current clamp. Passive membrane properties, including resting membrane potential, membrane time constant, input resistance, and capacitance were measured from small hyperpolarizing current steps (10 pA, 500 ms duration), from a zero-current level. Active properties were determined from a series of depolarizing current steps (0 to + 400 pA, 500 ms) from − 70 mV, maintained by addition of bias current. AP properties were determined from the first, second or fifth AP elicited at rheobase. Stimulation of the Schaffer collateral (SC) and temporoammonic (TA) pathways were made with a bipolar twisted Ni:Chrome wire electrode placed in either *str. radiatum* or *str. lacunosum-moleculare* in distal CA1. In all stimulation slices, CA3 was severed to prevent recurrent activation from antidromic activation of CA3. To assess synaptic strength of afferent inputs, 2 × stimuli of 200 µs duration (50 ms interval) were delivered at 10-s intervals at 30 V, 60 V, and 90 V levels from a constant-voltage stimulation box (Digitimer, Cambridge, UK). Recordings were first performed in cell-attached mode to identify cell spike output. Following breakthrough into whole-cell mode, EPSPs were recorded in current-clamp configuration with membrane potential biased to − 70 mV. All recordings were filtered online at 10 kHz with the built-in 4-pole Bessel filter and digitized at 20 kHz (Digidata1440, Molecular Devices, CA, USA). Traces were recorded in pCLAMP 9 (Molecular Devices, CA, USA) and stored on a personal computer. Analysis of electrophysiological data was performed offline using the open-source software package Stimfit [51], blind to both genotype and treatment conditions.

### Axon initial segment labelling and neuron visualization

Additional ex vivo brain slices were collected during preparation of tissue for ex vivo recordings (see above) and fixed for 1 h at room temperature in 4% paraformaldehyde in 0.1 M phosphate buffer (PB). Following fixation, slices were transferred to 0.1 M PB + 0.9% saline (PBS) and stored for up to 1 week. Immunohistochemistry was performed as previously described [52]. Briefly, slices were rinsed 3–4 times in PBS, then blocked in a solution containing 10% normal goat serum, 0.3% Triton X-100 and 0.05% NaN_3_ diluted in PBS for 1 h. Primary antibodies raised against AnkyrinG (1:1000; 75–146, NeuroMab, USA) and NeuN (1:1000, Millipore EMD, UK) were applied in a solution containing 5% normal goat serum, 0.3% Triton X-100 and 0.05% NaN_3_ diluted in PBS, for 24–72 h at 4 °C. Slices were then washed in PBS and secondary antibodies (AlexaFluor 488 and AlexaFluor 633, Invitrogen, UK, both 1:500) were applied in a solution containing 3% normal goat serum, 0.1% Triton X-100 and 0.05% NaN_3_ overnight at 4 °C. Slices were rinsed in PBS, desalted in PB and mounted on glass slides with Vectashield Hard-Set mounting medium (Vector Labs, UK). Stacks of images of the lower *str. pyramidale* upper *str. oriens* were acquired on a Zeiss LSM800 laser scanning confocal microscope, under a 60x (1.2 NA) objective lens at 2048 × 2048 resolution, with a step size of 0.25 µm. Axon Initial Segment (AIS) lengths were measured offline using ImageJ as segmented lines covering the full extent of AnkyrinG labelling observed. A minimum of 25 AISs were measured for each rat.

For CA1 pyramidal neuron reconstructions, fixed slices containing recorded neurons were fixed overnight in 4% paraformaldehyde + 0.1 M PB at 4 °C. Slices were then transferred to 0.1 M PB and stored until processing. For visualization, slices were washed 2–3 times in 0.1 M PB and then transferred to a solution containing Streptavidin conjugated to AlexaFluor568 (1:500, Invitrogen, UK) and 0.3% Triton X-100 and 0.05% NaN_3_. Slices were then incubated for 48–72 h at 4 °C. Slices were then washed in 0.1 M PB and mounted on glass slides with an aqueous mounting medium (VectaShield, Vector Labs, UK) and cover slipped. Neurons were imaged on an upright confocal (as above) with image stacks collected with a 20 × objective lens (2048 × 2048, 1 µm steps). Neurons were reconstructed with the SNT toolbox for FIJI/ImageJ [53], Sholl analysis performed, and branch lengths measured for the different dendritic compartments.

Dendritic protrusion analysis was performed on short dendritic segments (secondary dendrites) that were imaged with a 63 × objective lens (2.4 × digital zoom, 2048 × 2048 resolution giving 40 nm pixels, 0.13 µm z-step). These images were deconvolved (Huygens Software Package, Scientific Volume Imaging, The Netherlands), then dendritic protrusions counted as a function of length in FIJI. To estimate the total number of dendritic protrusions per dendritic compartment, the density was multiplied by the total length of dendrites in that compartment.

### Statistical analyses

#### In vivo* electrophysiology*

We compared the firing properties of the identified pyramidal neurons across days and sessions in WT and *Fmr1*^−/*y*^ rats in two ways. First, we modelled our data using a generalized linear mixed model (GLMM) approach in order to take into account the hierarchy of dependency in our data sets (genotypes-rats-neurons) and account for random effects. Second, we analysed the neuronal properties at the rat level by calculating the average value for each property across the neurons recorded in each rat [for additional discussion of the choice of statistical approach, see [53, 54]]. The results for GLMM analyses are presented in the main manuscript, while the results from the rat level analysis are presented in supplementary figure legends.

Statistical modelling routines for the linear mixed effects (LME) models were written and run using RStudio 1.0.153 (RStudio Team, 2016). Depending on the data distribution, linear mixed models (LMMs) or generalized linear mixed models (GLMMs) were fitted to single unit data metrics using the R package lme4 v1.1–17 [55]. Animal and cell identity (cluster number) were included in models as random effects, and the variables (terms) of interest (genotype, day, session-in-day) along with all interactions between them were included as fixed effects. Interactions and terms are progressively eliminated when a simpler model (i.e. a model not containing that term or interaction) fits the data equally well (based on likelihood ratio test). Consequently, the *p*-values reported in the context of LMEs are given by likelihood ratio tests between a model containing the variable or interaction in question and a model without that variable or interaction (a reduced/null model). When significant interactions were indicated by the LME, *post hoc* tests for between- and within-subjects effects were conducted by comparing estimated marginal means with t-tests with a Tukey correction for multiple comparisons.

Pearson’s correlation coefficients (*r*) are bounded between [− 1, 1], and the sampling distribution for highly correlated variables is highly skewed. Therefore, in order to convert the distribution to normal, r values were transformed to (*z*) values using the *Fisher's z transformation*:

$$r = \left( {0.5} \right)\ln \frac{1 + r}{{1 - r}}$$

For analysis of the cellular data at the rat level, the mean value for all cells from a given rat was calculated for each measure and analysed using a three-way ANOVA (genotype (between subjects) x day x session (within-subjects, session nested within day)). This same three-way ANOVA model was also used to analyse oscillatory power in each band. The effects of velocity on power of each oscillatory band of interest (Additional file 1: Fig S10) were analysed using a three-way ANOVA (genotype (between subjects) x day x velocity (within-subjects, velocity nested within day)).

The normality of all rat means’ distributions (i.e. group/sessions/metric) was tested using Kolmogorov–Smirnov (KS) tests. Nearly all rat means’ distributions passed the normality test (Distributions that did not pass the KS normality test: Firing rate: WT-session 3, *Fmr1*^−/*y*^ -session 1; Burst Prob: *Fmr1*^−/*y*^ -session 4,6; Sparsity: *Fmr1*^−/*y*^ -session 6; Place field size: *Fmr1*^−/*y*^ -session 2,3,5; MVL sgamma: WT-session 1, *Fmr1*^−/*y*^ -session 3; Theta PWR: *Fmr1*^−/*y*^ -session 1). However, given the small size of the distributions (*n* = 7 for rats for both genotypes), any estimations of distribution type cannot be accurate. Given that fitting an LME model that does not contain random effects would be the same as an ANOVA, we proceeded to use ANOVA for these rat-level analyses, despite the isolated distributions that did not satisfy the assumption of normality. The alternative would involve using nonparametric tests; however, these do not allow for hierarchical structure of repeated factors (session-in-day).

Power spectrograms were tested across all frequencies for significance at a *p* < 0.05 level, using a nonparametric randomization test, corrected for multiple comparisons across frequencies [22, 56].

The effects of genotype and session on the percentage of phase locked cells by each oscillatory band (Fig. 8) were analysed by fitting a series of multiple logistic regression models. The process is equivalent to that of hypothesis testing using LME. For each dataset, a full multiple logistic regression model was fit to the data with genotype, session and genotype x session interaction, and parameters: Phaselock (yes/no) ~ Intercept + Genotype + Session + Genotype x Session (Model 1). The fit of Model 1 was compared using log-likelihood ratio (*G*^2^ test) to the fit of a reduced model that did not contain the interaction term: Phaselock ~ Intercept + Genotype + Session (Model 2). To explore main effects of genotype and session, two separate models were used: Phaselock ~ Intercept + Genotype (Model 3) and Phaselock ~ Intercept + Session (Model 4). Their fit was compared to the null hypothesis that the simplest (intercept-only) model is correct again using log-likelihood ratio (*G*^2^ test). For individual comparisons between sessions and genotypes, we used two-proportion *z-test* [50] with correction for false discovery rate (alpha = 0.05) with Benjamini–Hochberg procedure.

As the LME framework for analysis of circular data is still under development, for analysis of oscillatory phase preference (Fig. 9), we first used Harrison-Kanji test [57] to analyse effects of genotype and day, as well as the interaction between them, with neuron as the unit of measurement. For comparisons between genotypes and days, we used the Watson-Williams test [58].

Statistical evaluation of data is presented in figure legends, main manuscript and supplementary tables. Average ± SEM values are reported throughout the manuscript unless stated otherwise. Significance was set at *p* < 0.05. Statistical analysis was carried out in SPSS 16.0 (IBM), R v.3.4.4 (R Core Team, 2018) or MATLAB (CircStat MATLAB toolbox [59]), and graphs created in GraphPad (Prism 8).

#### Down-sampling analysis

We report below that CA1 pyramidal cells in WT (but not *Fmr1*^−/*y*^) rats showed experience-dependent changes in firing rate, as well as in spatial firing properties. To examine whether the experience-dependent changes in spatial coding were secondary to the experience-dependent changes in firing rate also observed in WT but not *Fmr1*^−/*y*^ rats, we performed a bootstrapping-like down-sampling analysis. We sampled uniformly at random, 30 cells from each day and each genotype, 1000 times, with replacement. The size of subsamples was chosen to approximate the average cell number per rat at each session. Each of the subsamples was constrained to have an average firing rate that is equal to the overall mean firing rate of the WT cell population on day 2 (+ / − 5%). For each subpopulation of each genotype and day, we tested whether other metrics taken at the cell level (burst probability, spatial information, sparsity, place field size, % active bins, mean vector lengths relative to theta and gamma oscillations, and preferred theta phase) were statistically different from their overall population (two-sample t-test except for preferred theta phase). The portion of subpopulations that were statistically different from their overall population in each measure was used as the probability of the measure in question to be driven by changes in mean firing rate for the population tested (*p* < 0.05 indicating no modulation from mean firing rate; Additional file 2: Table S8).

For each subpopulation, we calculated the mean for each measure. These means (*n* = 1000) formed the distributions plotted in Additional file 1: Fig. S12.

Similarly, to control for the effect of mean firing rate change on firing rate map stability between the last session of day 1 (session 3) and the first session of day 2 (session 4), we performed two down-sampling analyses. For the first, the subsamples from both WT and *Fmr1*^−/*y*^ cells had to have an average firing rate decrease that is equal to the average firing rate decrease of WT cell population between session 3 and session 4 (+ / − 5%). For the second down-sampling, the subsamples from both WT and *Fmr1*^−/*y*^ cells had to have no change in firing rate (+ / − 5% of WT). As described above, the portion of 1000 subpopulations of each genotype that were statistically different from the overall population distribution was used as the probability of firing rate correlations to be driven by changes in mean firing rate for the population tested (*p* < 0.05 indicating no modulation from mean firing rate; Additional file 2: Table S8).

#### Ex vivo electrophysiology and anatomical analysis

Data generated from ex vivo brain slices were analysed as described previously [12]. Group sizes were chosen based on a presumed effect size of 15% and an overall statistical power of 80% (*N* = 7–8/group). For assessment of genotype effect, data were analysed using an LME based approach (see above), with animal and slice identity included as random effects. The *p*-value was then approximated using the Wald test, with effect size and variation estimated from the LMEs fitted to the data. For synaptic stimulation experiments, either the amplitude of the EPSP or the spike-probability were plotted per slice and compared using a 2-way ANOVA. If a genotype/stimulus interaction was observed, then Sidak *post hoc* tests were performed (corrected for multiple comparisons). For AIS lengths and morphology analysis, an LME analysis was performed, again using animal and slice identity as random effects with *p*-value approximated using the Wald test. For dendritic protrusion analysis, the animal average was analysed with Student’s t-test.

## Results

We recorded from the dorsal CA1 of adult *Fmr1*^−/*y*^ and WT rats as they foraged for randomly scattered cereal rewards in an initially novel cylindrical environment for three 10-min sessions (10 min ISI) over each of two consecutive days (Fig. 1A). As neural activity can be affected by ambulatory behaviour [60, 61], we first analysed the total path length of the rats in each of the three sessions across the two days (Fig. 1B). A mixed three-way ANOVA (genotype (between subjects) x day (within subjects) x session-within-day (within subjects, nested by session)) revealed no significant difference in mean path length between WT and *Fmr1*^−/*y*^ rats (genotype *F*_(1,12)_ = 0.695, *p* = 0.421), or between days (day *F*_(1,12)_ = 3.982, *p* = 0.069), but there was a significant decrease in path length across the three sessions within each day (session-in-day *F*_(2,24)_ = 51.97, *p* < 0.0001), with no significant interactions. This decrease in path length across sessions within each day may reflect habituation to the environment and/or decreased motivation to forage for food as the rat becomes more satiated. We also assessed whether the proportion of the environment explored by the rats differed between genotypes or across days and sessions. Rats from both genotypes explored more than 93% of the environment during every session (Fig. 1C). However, there was a small but significant difference between WT and *Fmr1*^−/*y*^ rats (3-way ANOVA, main effect of genotype *F*_(1,12)_ = 17.03, *p* = 0.001) whereby the *Fmr1*^−/*y*^ rats visited more of the environment than WT rats. There was also an overall difference between sessions within each day (session-in-day effect *F*_(2,24)_ = 13.46, *p* < 0.001) with slightly less of the environment being explored on later sessions than earlier sessions each day, but there were no significant differences in exploration across days (*F*_(1,12)_ = 0.267, *p* = 0.615), and no significant interactions (*p*’s > 0.05). As there was a difference between genotypes in the proportion of the environment that was explored during each session, we considered whether this might contribute to any of the differential changes in pyramidal neuron activity and spatial firing properties between groups presented below (see discussion of each finding). Finally, as speed of movement can influence both pyramidal neuron activity and oscillatory activity, we also analysed the amount of time during each session the rats spent moving at different speeds (Additional file 1: Fig. S1). A four-way ANOVA (genotype, day, session-in-day, velocity bin) revealed no significant differences between genotypes (*p* = 0.163), nor any significant 2-, 3- or 4-way interactions including genotype and velocity bin, indicating that the velocity profile did not differ significantly between genotypes (*p*’s > 0.05).

### Experience-dependent decrease in CA1 pyramidal neuron activity is attenuated in *Fmr1*^−/*y*^ rats

**Fig. 2:**
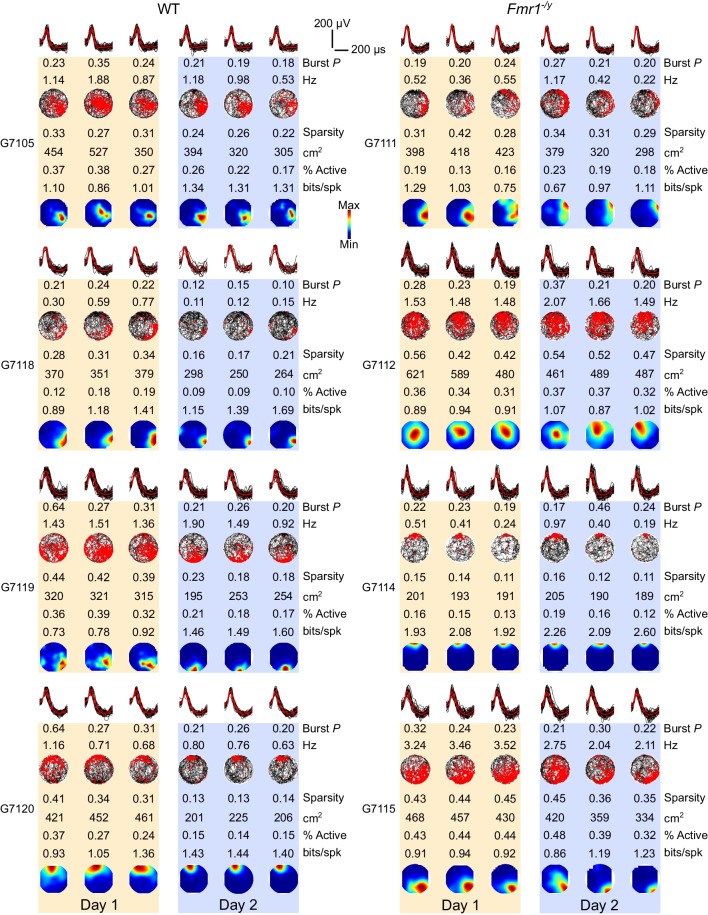
Example firing rate maps and activity measures. Example firing rate maps from 4 WT (left) and 4 *Fmr1*^−/*y*^ (right) CA1 pyramidal cells, each from a different animal. *Top:* Spike waveforms (black lines) from the tetrode channel with the highest amplitude waveforms across the 6 recording sessions. Red solid lines indicate the mean waveforms. Dotted red lines indicate the standard error. *Middle:* movement trajectory (black path) and superimposed action potentials of the cell (red dots) across the 6 sessions in the novel environment. Mean session firing rates (Hz) and burst probability (Burst *P*) are stated above each plot. *Bottom:* Smoothed firing rate maps of the same cell, with warmer colours indicating higher firing rates. Spatial information (bits/spk), sparsity, place field size (cm^2^) and percentage of visited bins in which the cells fired (% Active) for each session is stated above the firing rate map. Pale yellow and pale purple backgrounds denote data from Day 1 and Day 2, respectively

Over the duration of the experiment, we recorded 288 neurons from WT and 246 from *Fmr1*^−/*y*^ rats that were sufficiently isolated (based on L-ratio and Isolation D; for details see Methods), were classified as pyramidal neurons (spike width > 250 μs and mean firing rate < 5 Hz), and were active in the environment (mean firing rate > 0.1 Hz) (Additional file 1: Tables S1-2 show numbers of pyramidal neurons recorded from each rat). Histological analysis confirmed that the tetrodes were in the CA1 cell layer of the dorsal hippocampus in all rats and were clustered approximately midway along the proximal–distal axis between CA3 and subiculum (Additional file 1: Fig. S1). Figure 2 depicts examples of four active CA1 pyramidal cells from each genotype recorded across the 6 sessions, each from a different rat, illustrating the average waveforms for each cell across the 6 sessions, as well as their mean firing rates, burst probabilities, spatial firing rate maps, and measures of spatial firing.

**Fig. 3:**
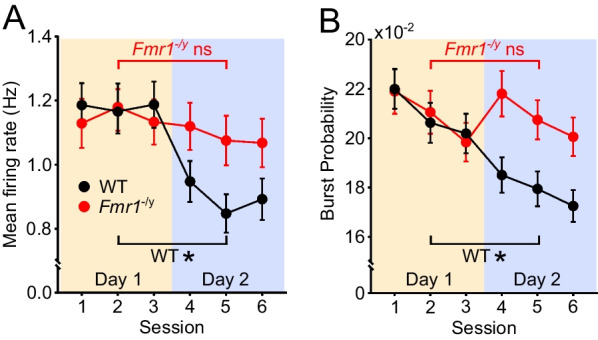
WT but not *Fmr1*^−/*y*^ CA1 pyramidal neurons exhibit experience-dependent changes in mean firing rate and burst probability. **A** The mean firing rate of CA1 pyramidal neurons decreased significantly between the first and second day of exploration in the novel environment in WT but not in *Fmr1*^−/*y*^ rats (genotype x day interaction *p* = 0.006; *post hoc* comparisons Day 1 vs Day 2, WT: *p* < 0.0001; *Fmr1*^−/*y*^: *p* = 0.131). There were no significant differences in mean firing rate between genotypes on either Day 1 or Day 2 (*post hoc* comparisons *p*’s > 0.05). **B** Burst probability of pyramidal neurons decreased significantly between Day 1 and Day 2 in WT, but not in *Fmr1*^−/*y*^ rats (genotype x day interaction *p* = 0.005; *post hoc* comparisons Day 1 vs Day 2: WT: *p* < 0.0001; *Fmr1*^−/*y*^* p* = 0.924). There were no significant differences between genotypes on either day (*post hoc* comparisons *p*’s > 0.05). Data represent cell means and SEMs. Statistical analyses used linear mixed effect (LME) modelling with genotype, day and session-in-day as fixed factors, cell and rat as random factors, followed by Tukey comparisons on emmeans for significant interactions. *N*_WT-D1_ = 222 cells, *N*_WT-D2_ = 207 cells, *N*_KO-D1_ = 211 cells, *N*_KO-D2_ = 205 cells. Pale yellow and pale purple backgrounds denote data from Day 1 and Day 2, respectively

CA1 pyramidal neurons have previously been reported to fire at higher rates in novel than in familiar environments [29, 62] and this phenomenon has been linked to enhanced input integration [60] during novelty. We therefore examined whether the mean firing rates of CA1 pyramidal neurons changed as a function of experience in the novel environment in our experiment, and whether this differed between WT and *Fmr1*^−/*y*^ rats. Figure 3A shows the mean firing rates of all the active CA1 pyramidal cells for each genotype in each session, while Additional file 1: Figure S3A shows violin plots of the mean firing rates of all cells in each session, and Additional file 1: Figure S3B shows box plots based on the mean firing rates at an animal (rather than individual cell) level. Linear mixed effects (LME) modelling analysis using genotype, day and session-within-day as fixed factors, and animal and cell as random factors, was used to statistically analyse the cell-level data. This revealed a significant genotype x day interaction (*p* = 0.006), with no significant additional contribution of session-in-day. Exploring the genotype x day interaction further, *post hoc* Tukey paired comparisons on emmeans comparing Day 1 and Day 2 for each genotype indicated that WT rats had significantly lower mean firing rates on Day 2 than Day 1 (*p* < 0.0001), while those of *Fmr1*^−/*y*^ rats did not differ significantly between days (*p* = 0.131). Between genotype *post hoc* Tukey comparisons on emmeans indicated no significant differences between WT and *Fmr1*^−/*y*^ on either day (Day 1: *p* = 0.992; Day 2, *p* = 0.130). Thus, the mean firing rates of WT and *Fmr1*^−/*y*^ CA1 pyramidal cells did not differ overall, but WT cells showed a significant experience-dependent decrease in firing between Day 1 and Day 2, whereas *Fmr1*^−/*y*^ cells did not.

To further explore pyramidal neuron activity in WT and *Fmr1*^−/*y*^ rats, we calculated their burst probability, which is a property of pyramidal neurons that is controlled by perisomatic and dendritic inhibition [41]. High burst probability is linked to plasticity processes underlying new place field formation [60, 61]. As shown in Fig. 3B (summary data; individual cell data Additional file 1: Fig. S3C, rat averages data in S3D), the burst probability of CA1 pyramidal neurons decreased significantly between days in WT but not in *Fmr1*^−/*y*^ rats (LME: genotype x day interaction *p* = 0.005; Tukey *post hoc* paired comparisons on emmeans Day 1 vs Day 2: WT: *p* < 0.0001; *Fmr1*^−/*y*^* p* = 0.924). There was no significant difference in burst probability between genotypes on either day (*post hoc* Tukey comparisons on emmeans comparing WT vs *Fmr1*^−/*y*^: Day 1 *p* = 0.973, Day 2 *p* = 0.068). There was also a significant decrease in burst probability across sessions within a day (LME: session-in-day effect *p* = 0.013), but this did not differ between genotypes (LME: genotype x session-in-day interaction *p* = 0.916). Additional file 2: Table S3 shows the full LME analyses for both mean firing rates and burst probabilities.

Overall, these analyses indicate that both the mean firing rate and the burst probability of WT CA1 pyramidal neurons decreases between Day 1 and Day 2 of exposure to a novel environment, whereas these experience-dependent changes are not seen in *Fmr1*^−/*y*^ neurons. It is unlikely that the differential pattern of firing rate and burst probability changes seen across days in WT and *Fmr1*^−/*y*^ rats are secondary to any differences in exploratory behaviour between genotypes, as while more of the environment was explored on both days by *Fmr1*^−/*y*^ rats, there were no changes in exploratory behaviour between Day 1 and Day 2 that could account for the changes in firing rate and burst probability seen in WT cells.

### Experience-dependent refinement of spatial tuning of CA1 pyramidal neurons is disrupted in *Fmr1*^−/*y*^ rats

While the mean firing rate of neurons typically decreases with experience in an environment, the spatial tuning of pyramidal neurons has been shown to increase as a function of experience both in mice [28, 63] and rats [64], with the biggest increase observed between the first and second day of exposure in an initially novel environment [28, 64]. We therefore assessed how the spatial firing properties of the CA1 pyramidal neurons from *Fmr1*^−/*y*^ and WT rats change over repeated sessions in the novel environment. The spatial tuning of a pyramidal neuron can be quantified and expressed as a number of metrics such as spatial information, sparsity, place field size, and the proportion of the environment in which the neuron fires. Spatial information measures the amount of information a single action potential conveys about the animal’s location in an environment, with higher spatial information reflecting more informative firing [44]. Sparsity is a measure that reflects how compact a place field is, with lower sparsity reflecting more compact firing [45]; Place field size is calculated from the firing rate map and is a measure of the area covered by the cell’s place field, with smaller place fields reflecting more selective spatial firing. This is closely related to the % active pixels measure, which is the proportion of visited pixels in the environment in which the neuron is active. Firing in fewer pixels reflects more spatially selective firing.

**Fig. 4:**
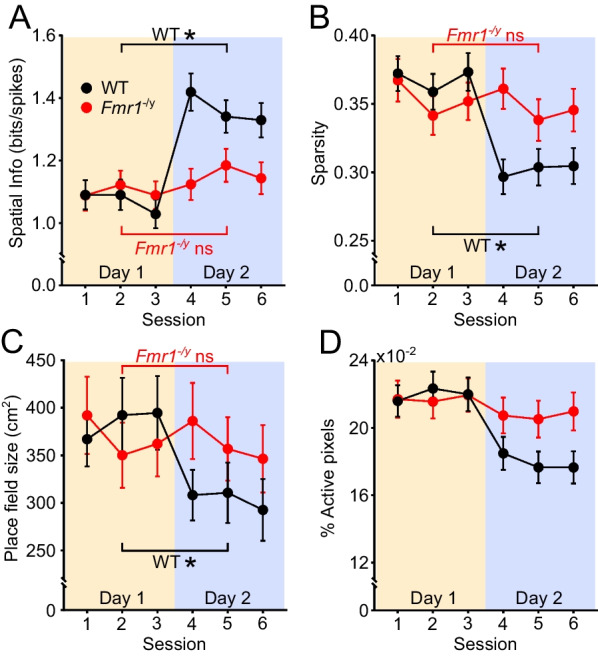
Experience-dependent refinement of spatial coding in the CA1 pyramidal cells is impaired in *Fmr1*^−/*y*^ rats. **A** The spatial information of CA1 pyramidal cells increased between the first and second day of exploration in the novel environment in WT but not in in *Fmr1*^−/*y*^ rats, with no significant difference between genotypes on either day (genotype x day interaction *p* = 0.005; *post hoc* comparisons Day 1 vs Day 2: WT *p* < 0.001; *Fmr1*^−/*y*^* p* = 0. 129; WT vs *Fmr1*^−/*y*^: *p* > 0.05 on both days). **B** The spatial sparsity of CA1 pyramidal cell firing decreased significantly between Day 1 and Day 2 in WT but not *Fmr1*^−/*y*^ rats, with no differences between genotypes on either day (genotype x day interaction *p* = 0.013; *post hoc* comparisons Day 1 vs Day 2: WT *p* < 0.001; *Fmr1*^−/*y*^* p* = 0.091; WT vs *Fmr1*^−/*y*^: *p* > 0.05 on both days). **C** The size of CA1 pyramidal cell place fields decreased significantly between Day 1 and Day 2 in WT but not *Fmr1*^−/*y*^ rats, with no differences between genotypes on either day (genotype x day interaction *p* = 0.017; *post hoc* comparisons Day 1 vs Day 2: WT *p* < 0.001; *Fmr1*^−/*y*^* p* = 0.051; WT vs *Fmr1*^−/*y*^: *p* > 0.05 on both days). **D** The proportion of visited pixels in which CA1 pyramidal cells fired (% active bins) did not differ significantly across days or between genotypes (genotype x day interaction *p* = 0.070). Data represent cell means and SEMs. Statistical analyses using LME modelling and Tukey *post hoc* tests as in Fig. 2. *N*_WT-D1_ = 222, *N*_WT-D2_ = 207, *N*_KO-D1_ = 211, *N*_KO-D2_ = 205. Pale yellow and pale purple backgrounds denote data from Day 1 and Day 2, respectively

The example cells in Fig. 2 include firing rate maps across the six sessions, with spatial information, sparsity, place field size and % active pixel indicated for each example cell. As can be seen from these examples, spatial tuning appeared to be enhanced in WT cells on Day 2 relative to Day 1, whereas this was less apparent in the *Fmr1*^−/*y*^ cells. LME models were used to statistically analyse each of these measures of spatial activity across the population of active pyramidal cells in each session (fixed factors: genotype, session, session-in-day; random factors: animal, cell). CA1 pyramidal cells in WT rats showed a significant increase in spatial information between the first and second day of exposure to the novel environment. However, cells in *Fmr1*^−/*y*^ rats did not show this increase (Fig. 4A; LME: genotype x day interaction *p* < 0.005; *post hoc* Tukey paired comparisons on emmeans between Day 1 and Day 2: WT *p* < 0.001, *Fmr1*^−/*y*^* p* = 0.129. There were no significant differences between genotypes on either day (*post hoc* Tukey comparisons between emmeans: Day 1 *p* = 0.9304; Day 2 *p* = 0.079).

Both sparsity (Fig. 4B) and place field size (4C) also decreased significantly between Day 1 and Day 2 in WT cells but not in *Fmr1*^−/*y*^ cells, again consistent with experience-dependent refinement of spatial tuning in WT rats that is absent in *Fmr1*^−/*y*^ rats (LME Sparsity: genotype x day interaction *p* = 0.013; Tukey *post hoc* WT: Day 1 vs Day 2 *p* < 0.001, *Fmr1*^−/*y*^: Day 1 vs Day 2 *p* = 0.091. LME Place field size genotype x day interaction *p* = 0.017; Tukey *post hoc* WT: Day1vsDay2 *p* < 0.001, *Fmr1*^−/*y*^: Day 1 vs Day 2 *p* = 0.513). Similar to the pattern for spatial information, there were no significant differences between genotypes on either day for either sparsity (Tukey *post hoc* Day 1: WT vs *Fmr1*^−/*y*^* p* = 0.973, Day 2: WT vs *Fmr1*^−/*y*^* p* = 0.094) or place field size (Tukey *post hoc* Day 1: WT vs *Fmr1*^−/*y*^* p* = 0.927, Day 2: WT vs *Fmr1*^−/*y*^* p* = 0.503). The fourth measure of spatial tuning, the proportion of visited pixels in which a cell fired (Fig. 4D) showed a similar pattern to the other measures of spatial tuning, but the LME analysis revealed a significant main effect of Day (LME day *p* < 0.001), but no significant genotype x day interaction (*p* = 0.070), suggesting that by this measure, spatial tuning improved similarly in both genotypes across days. The individual cell and rat mean data for each of these spatial measures are depicted in Additional file 1: Figure S4, and the LME statistics in Additional file 2: Table S4.

Taken together, these data indicate that CA1 pyramidal neurons from WT rats exhibit experience-dependent refinement of spatial tuning between the first and second day of exploring a novel environment, but that this refinement is attenuated in *Fmr1*^−/*y*^ rats. It is unlikely that this pattern of results can be explained by differences in exploratory behaviour between WT and *Fmr1*^−/*y*^ rats. Specifically, while the calculation of spatial information, sparsity and % active pixels are all influenced by the extent of exploration, we would expect this to result in higher spatial information together with lower sparsity and lower % active bins in the *Fmr1*^−/*y*^ rats which explored more of the environment than WT. This is the opposite from what was found on Day 2. The calculation of place field size is independent of visited pixels, and so it too is unlikely to be influenced by the small differences between genotypes in the number of visited pixels. Finally, as there were no differences between the amount of the environment explored on Day 1 and Day 2, it is unlikely that differences in exploration could account for the changes in spatial tuning across days in WT (but not in *Fmr1*^−/*y*^) cells.

We also considered whether the enhanced spatial tuning on day 2 in WT but not in *Fmr1*^−/*y*^ cells could be secondary to the decreased mean firing rate of WT cells on Day 2. To address this, we conducted a bootstrapping-like downsampling analysis. We sampled randomly 30 cells from each day and each genotype, 1000 times. The spatial information, sparsity, place field size and % active bins were extracted only for 30-cell subpopulations with average mean firing rate equal to the overall mean firing rate of the WT cell population on day 2 (+ / − 5%). The portion of 30-cell subpopulations (out of 1000) that were statistically different from their overall population in a given measure was used as the probability of that measure to be modulated by mean firing rate for that population. Our downsampling analysis indicated that the experience-dependent increase in the spatial tuning of WT is not secondary to the decrease in mean firing rate (Additional file 1: Fig S12; Additional file 2: Table S8).

### The stability of CA1 pyramidal neuron firing rate maps does not differ between WT and *Fmr1*^−/*y*^ rats

**Fig. 5:**
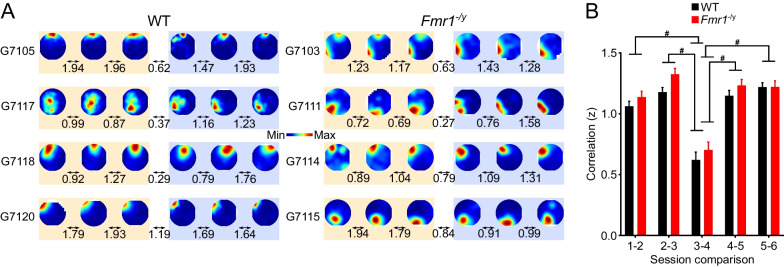
The stability of CA1 pyramidal cell firing rate maps does not differ between WT and *Fmr1*^−/*y*^ rats. **A** Example firing rate maps from 4 WT (left) and 4 *Fmr1*^−/*y*^ (right) CA1 pyramidal cells, each from a different animal. Smoothed firing rate maps of the same cell, with warmer colours indicating higher firing rates. R values from Pearson correlations (Fisher z-transformed) between the firing rate maps of consecutive exploration sessions are indicated below the arrows between sessions. **B** Mean Pearson correlation coefficients (Fisher z-transformed) between firing rate maps for consecutive sessions for the population of WT and *Fmr1*^−/*y*^ pyramidal cells. Firing rate map stability did not differ significantly between WT and *Fmr1*^−/*y*^ cells (genotype *p* = 0.094). Both genotypes had less stable maps between days (Session 3–4 comparison) than between sessions of the same day (*post hoc* tests on S3-S4 vs all other comparisons, ^#^*p*’s < 0.05). Data represent cell means and SEM. Statistical analyses used LME modelling with genotype and session comparison (i.e. S1-S2, S2-S3, S3-S4, S4-S5 and S5-S6) as fixed factors, and cell and rat as random factors, followed by Tukey *post hoc* comparisons on emmeans for significant main effect of session. S1vsS2: *N*_WT_ = 194, *N*_KO_ = 167, S2vsS3: *N*_WT_ = 191, *N*_KO_ = 179, S3vsS4: *N*_WT_ = 121, *N*_KO_ = 152, S4vsS5: *N*_WT_ = 190, *N*_KO_ = 161, S5vsS6: *N*_WT_ = 188, *N*_KO_ = 169. Pale yellow and pale purple backgrounds denote data from Day 1 and Day 2, respectively

Another key property of pyramidal neurons that has been linked to synaptic plasticity is the stability of their firing rate maps [33]. Interestingly, the plasticity mechanisms behind this property are molecularly dissociable from those that govern the experience-dependent increase of spatial information [28]. To assess firing rate map stability, we calculated the correlation between the firing rate maps across consecutive sessions (yielding 5 session comparisons: S1-S2, S2-S3, S3-S4, S4-S5 and S5-S6)). Our analysis included only pyramidal neurons with spatial information > 0.5 bits/spike in both sessions within a pair, to ensure that low correlations were not a result of poor spatial coding [65, 66]. Firing rate maps from example cells are depicted in Fig. 5A, and the summary data of the mean correlations between consecutive sessions for each genotype are shown in Fig. 5B. Pyramidal neurons from *Fmr1*^−/*y*^ and WT rats had similar spatial firing rate map correlations between consecutive sessions, with no significant differences between genotypes (LME main effect of genotype *p* = 0.094). There was a significant main effect of session comparison (LME main effect of comparison *p* < 0.001), driven by both groups showing lower correlations between days (S3-S4) than between sessions within each day (Tukey *post hoc* S3-S4 vs S1-S2, S2-S3, S4-S5 and S5-S6 all *p*’s < 0.05; there were no significant differences between any other session comparisons). The interaction between genotype and comparison was also not significant (LME genotype x session comparison *p* = 0.617). Individual cell data and rat average data are shown in Additional file 1: Figure S5 and LME analyses in Additional file 2: Table S5.

To address whether the decrease in mean firing rate in WT cells between Day 1 and Day 2 may have contributed to the relatively low stability between S3 and S4, we conducted a similar bootstrapping-like downsampling procedure as described above, whereby we randomly sampled 30 cells from both WT and *Fmr1*^−/*y*^ S3-S4 distributions 1000 times. Only 30-cell subpopulations with average firing rate S3-S4 difference equal to the mean of WT cells were used. The proportion of subpopulations with S3-S4 correlations statistically different from their entire population was used as the probability for the firing rate map correlations between S3 and S4 to be modulated by mean firing rate. We repeated the downsampling, but this time only 30-cell subpopulations with null average firing rate S3-S4 difference were used. We found that the low stability of firing rate maps between S3 and S4 was not due to the decrease of firing rate in WT cells, and that *Fmr1*^−/*y*^ show similar firing rate map stability to WT cells even if there is no difference in their mean firing rate decrease.

These analyses indicate that pyramidal neurons from *Fmr1*^−/*y*^ rats exhibit normal spatial stability in their firing rate maps, both between sessions within the same day and between the first two days of exposure to a novel environment. While this is consistent with stable spatial coding in *Fmr1*^−/*y*^ rats, this finding contrasts with the observed deficits in the experience-dependent improvement in spatial tuning over the two days.

### *Fmr1*^−/*y*^ CA1 pyramidal neurons are intrinsically more excitable but receive less synaptic input from the medial entorhinal cortex

**Fig. 6:**
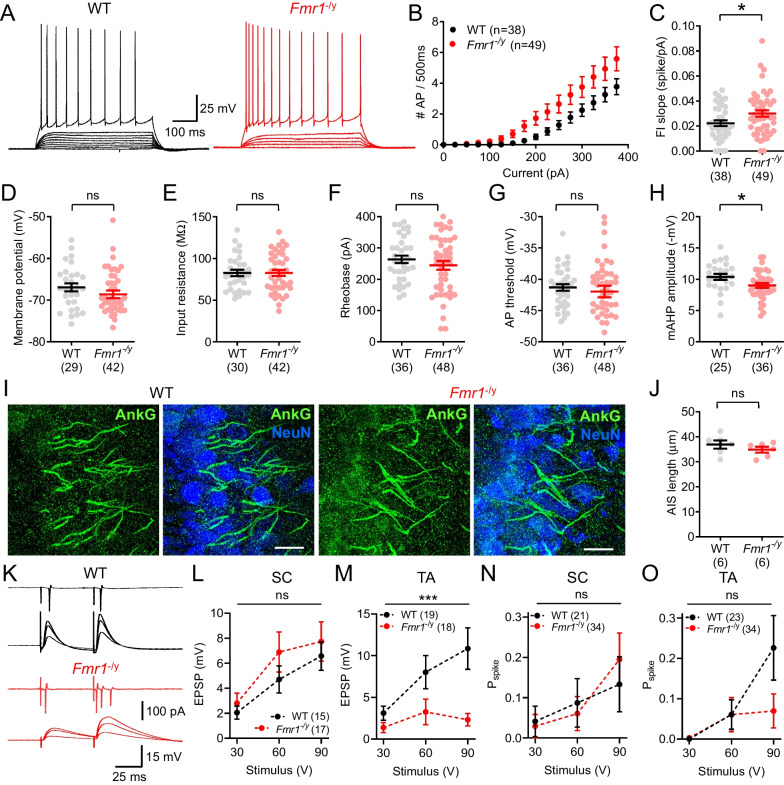
*Fmr1*^−/*y*^ CA1 pyramidal neurons display increased excitability, which correlates with reduced synaptic inputs from the medial entorhinal cortex. **A** Representative traces from CA1 pyramidal neurons in the dorsal hippocampus from WT and *Fmr1*^−/*y*^ rats, in response to depolarizing current injections (0–400 pA, 25 pA steps, 500 ms duration). **B** Action potential discharge in 500 ms compared to injected current for all recorded CA1 pyramidal neurons in WT and *Fmr1*^−/*y*^ rats. Data shown as the mean response recorded per rat, with total number of neurons indicated. **C** The slope of the curve in (**B**) quantified for each neuron. Individual neuron data are shown overlain as filled circles, and the number of tested neurons shown below in parentheses. Average resting membrane potential (**D**) and input resistance (**E**), measured from the zero-current potential. Average data are plotted for the rheobase current (**F**), the voltage threshold (**G**), and medium afterhyperpolarization (mAHP) amplitude (**H**), the latter two measured from the first action potential at rheobase. **I** Visualization of the AIS in flattened confocal z-stacks after immunofluorescent labelling for AnkyrinG (AnkG, green pseudocolour) and merged with NeuN (blue pseudocolour) from WT and *Fmr1*^−/*y*^ rats. Scale bars shown: 20 µm. **J** Quantification of AIS length, displayed as the average of individual animals. **K** Representative traces from cell attached recordings (upper traces) and whole cell recordings (lower traces) following stimulation of str. lacunosum moleculare to distal CA1, from WT and *Fmr1*^−/*y*^ rats. EPSP data is shown as the average traces in response to stimulation intensities. **L** Average data for EPSP amplitude in response to the same stimulation intensities, delivered to the Schaffer collateral (SC), and **M** temporoammonic (TA) pathways. Average spike probability, measured in cell-attached recordings, for SC (**N**) and TA (**O**) paths. For **L**–**O**, all graphs display the result from 2-way ANOVA for genotype shown above the chart and number of tested neurons indicated in parentheses. All data is shown as mean ± SEM. Statistics shown as: ns—*p* > 0.05, *—*p* < 0.05, and ***—*p* < 0.001 from GLMM analysis, except panels **B**, **K**, **L**, **M**, **N** which are the result of 2-way ANOVA for genotype

Overall, our analyses suggest that loss of FMRP leads to reduced adaptation of CA1 pyramidal neurons to a novel environment across days, maintaining high spike discharge and low spatial tuning independent of experience. A key question is whether these phenotypes result from changes in the intrinsic properties of CA1 pyramidal neurons themselves, alterations in the inputs they receive, or a combination of the two. To address this question, we performed whole-cell patch clamp recordings from adult CA1 pyramidal neurons (WT: 36 cells from 10 rats, *Fmr1*^−/*y*^: 48 cells from 12 rats) from the dorsal hippocampus matched to the location of the in vivo recordings. First, we measured the intrinsic excitability of CA1 pyramidal neurons in response to depolarizing current steps (0–400 pA, 25 pA steps, 500 ms duration), which in WT neurons resulted in repetitive action potential discharge (Fig. 6A). The same stimuli delivered to CA1 pyramidal neurons from *Fmr1*^−/*y*^ rats resulted in consistently increased AP discharge over the range of currents tested (Fig. 6B). Comparison of the slopes of individual action potential discharge to increasing current revealed an increase in the overall slope in *Fmr1*^−/*y*^ CA1 pyramidal neurons, compared to WT (Additional file 2: Table S6; LME: *p* = 0.04). We have recently shown in the CA1 of juvenile *Fmr1*^−/*y*^ mice that such changes in intrinsic cell excitability are correlated with altered membrane potential, reduced threshold to fire, and longer AIS [12]. Comparing CA1 pyramidal neurons between WT and *Fmr1*^−/*y*^ rats, we observed no difference in passive properties such as resting membrane potential (Fig. 6C; LME: *p* = 0.45), input resistance (Fig. 6D; LME: *p* = 0.51), rheobase current (Fig. 6E, LME: *p* = 0.18) or action potential threshold (Fig. 6F; LME: *p* = 0.46). However, the medium afterhyperpolarization (mAHP) was reduced by 13% in *Fmr1*^−/*y*^ CA1 pyramidal neurons compared to WT (Fig. 6G, LME: *p* = 0.05). Consistent with the similar action potential thresholds measured between genotypes, we observed no difference in the AIS length, as assessed by AnkyrinG immunostaining (Fig. 6H), between *Fmr1*^−/*y*^ and WT rats (Fig. 6I; LME: *p* = 0.33).

We next examined the synaptic strength of the two major excitatory inputs to CA1: the Schaffer collateral (SC) inputs from CA3 neurons in *stratum radiatum* and *oriens* and the temporoammonic (TA) inputs from layer 3 of the medial entorhinal cortex (MEC3) onto the distal dendrites in *stratum lacunosum-moleculare* [67]. We have previously shown reduced strength of TA synapses with CA1 pyramidal neurons in the mouse model of FXS [12]. Cell attached and whole cell recordings from CA1 pyramidal neurons were performed in the presence of 50μΜ picrotoxin to block inhibitory neurotransmission (Fig. 6J and Additional file 1: Fig. S6 A&B). In whole-cell patch clamp recordings, stimulation of SC afferents in *stratum radiatum* did not show a difference between genotypes in the resulting excitatory postsynaptic potential (EPSP) measured over the range of stimulation intensities tested (30, 60 and 90 V; Fig. 6K; 2-way ANOVA main effect of genotype *F*_(1,68)_ = 2.03, *p* = 0.16). In contrast, there was a robust decrease in the recruitment of EPSPs in CA1 pyramidal neurons from the *Fmr1*^−/*y*^ rats in response to TA stimulation compared to WT rats (Fig. 6L; 2-way ANOVA: main effect of genotype *F*_(1,98)_ = 15.4, *p* = 0.0002). This was reflected by a similar input–output slope for SC stimulation between genotypes, but a reduced slope for TA inputs in the *Fmr1*^−/*y*^ rats (Additional file 1: Fig. S6C, K-W_(4,50)_ = 16.12, *p* = 0.001 Kruskal–Wallis test). We also observed bidirectional effects on short-term plasticity in the different inputs. Specifically, at the SC inputs paired-pulse ratio (PPR) was lower in slices from *Fmr1*^−/*y*^ than from those of WT rats at all stimulation intensities (Additional file 1: Fig. S6D; 2-way ANOVA: main effect of genotype *F*_(1,54)_ = 10.6, *p* = 0.002), whereas at the TA inputs PPR was higher in the *Fmr1*^−/*y*^ rats at the 90 V stimulation intensity (Additional file 1: Fig. S6E; 2-way ANOVA genotype x stimulus interaction *F*_(2,85)_ = 3.92, *p* = 0.024, 90 V: t_(13,14)_ = 2.47, *p* = 0.05). These reductions in synaptic input were not associated with gross anatomical changes in CA1 pyramidal neurons (Additional file 1: Fig. S7A), as Sholl analysis of recorded neurons displayed no difference in branching pattern (Additional file 1: Fig. S7A). Furthermore, there was no difference in total or compartment specific dendritic lengths between genotypes (Additional file 1: Fig. S7C-F). Finally, there was a tendency towards reduced dendritic protrusions in the distal apical dendrites, consistent with a reduction in synaptic number at TA inputs, which was not apparent at dendrites aligned to SC afferents (Additional file 1: Fig. S8).

To determine whether the increased AP discharge observed in CA1 pyramidal neurons is sufficient to overcome the reduced synaptic strength observed in response to electrical stimulation of the TA pathway, we next asked if spike output was altered. In cell-attached recordings from pyramidal neurons under the same conditions, we performed paired-pulse stimulation at SC and TA synapses to CA1. We observed a similar recruitment of CA1 pyramidal neurons to SC pathways stimulation in *Fmr1*^−/*y*^ and WTs, both in terms of spike probability (Fig. 6M; 2-way ANOVA: main effect of genotype *F*_(1,156)_ = 0.032, *p* = 0.86) and in the number of cells that produced a spike in any response to any stimulus (Additional file 1: Fig. S6F; *χ*^2^ = 0.30, *p* = 0.59). In contrast, following TA afferent stimulation, the overall spike probability was not different between genotypes at 30 V and 60 V stimulation (Fig. 6N; Additional file 2: Table S6; LME: *p* = 0.99). However, it tended towards lower spiking in *Fmr1*^−/*y*^ than in WT rats at 90 V (Additional file 2: Table S6; LME: *p* = 0.06), suggesting a level of compensation, at least at lower stimulation intensities (2-way ANOVA: main effect of genotype *F*_(1,163)_ = 2.276, *p* = 0.13). Nevertheless, the overall recruitment of individual CA1 pyramidal neurons was reduced by approximately threefold (Additional file 1: Fig. S6 *F*; *χ*^2^ = 4.37, *p* = 0.04), indicating a degree of information loss. Despite the reduction in CA1 pyramidal neuron recruitment, when present, spikes showed a similar coefficient of variation of spike times between genotypes, in the absence of GABA_A_ receptor mediated inhibition (Additional file 1: Fig. S6G; K-W_(4,44)_ = 0.23, *p* = 0.97).

Together, these data show that CA1 pyramidal neurons in the dorsal hippocampus of the *Fmr1*^−/*y*^ rat are hyperexcitable, which results from a decrease in medium AHP amplitude. This hyperexcitability is sufficient to overcome the reduced synaptic strength following TA stimulation received by *Fmr1*^−/*y*^ CA1 pyramidal neurons.

### Power of hippocampal theta and gamma oscillations does not differ significantly between WT and *Fmr1*^−/*y*^ rats

**Fig. 7:**
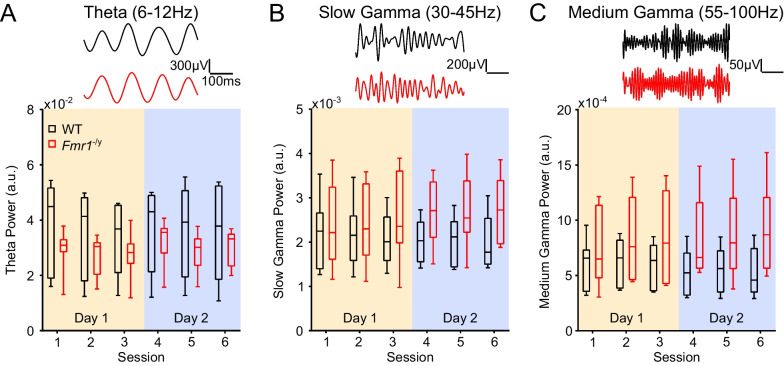
No differences between WT and *Fmr1*^−/*y*^ rats in the power of hippocampal oscillatory activity. **A** Top: Example CA1 LFP traces bandpass filtered for theta (6–12 Hz) from a WT (black) and an *Fmr1*^−/*y*^ (red) rat. Bottom: Box and whisker plots depicting theta power (6–12 Hz) for each session. The middle line represents rat median, upper and lower end of the box represents 95^th^ and 5^th^ percentile, whiskers represent maximum and minimum values. There was a significant decrease in theta power across sessions within a day (*p* = 0.021) but no significant difference between genotypes (*p* = 0.367) or days (*p* = 0.581) and no significant interactions. **B** Slow gamma (30–45 Hz) (same layout as in **A**) exhibited no significant differences between genotypes (*p* = 0.184), days (*p* = 0.735) or sessions within a day (*p* = 0.817) and no significant interactions. **C** Medium gamma (55–100 Hz) (same layout as in **A**) exhibited no significant differences between genotypes (*p* = 0.103), days (*p* = 0.521) or sessions within a day (*p* = 0.265) and no significant interactions. Statistical analysis used 3-way ANOVA (genotype, day, session in day). *N*_WT_ = 7, *N*_KO_ = 7. Pale yellow and pale purple backgrounds denote data from Day 1 and Day 2, respectively

The firing patterns of CA1 pyramidal neurons can be temporally organized by both theta and gamma oscillatory frequencies of local field potentials (LFP) while an animal explores its environment [68, 69]. The different frequency bands reflect hippocampal circuit organization as well as unique inputs, with slow gamma associated with inputs from CA3, and medium gamma with inputs from medial entorhinal cortex [49, 70] (Additional file 1: Fig. S9 A). Given the changes in MEC input strength observed in the ex vivo experiments described above, we first examined the mean power of oscillations in the theta (6-12 Hz, Fig. 7A), slow gamma (30-45 Hz, Fig. 7B) and medium gamma (55–100 Hz, Fig. 7C) ranges during periods when the rats were moving (> 3 cm/s) over the six sessions. Although both slow and medium gamma power appeared to be higher in the *Fmr1*^−/*y*^ rats, 3-way mixed ANOVAs (genotype, day, session-in-day) indicated that there were no significant differences between genotypes or days, and no genotype x day interactions for theta, slow gamma or medium gamma power (Theta: genotype *F*_(1,12)_ = 0.880, *p* = 0.367, day *F*_(1,12)_ = 0.322, *p* = 0.581, genotype x day *F*_(1,12)_ = 0.666, *p* = 0.430; Slow gamma: genotype *F*_(1,12)_ = 1.988, *p* = 0.184, day *F*_(1,12)_ = 0.120, *p* = 0.735, genotype x day *F*_(1,12)_ = 1.613, *p* = 0.228; Medium gamma: genotype *F*_(1,12)_ = 3.109, *p* = 0.103, day *F*_(1,12)_ = 0.437, *p* = 0.521, genotype x day *F*_(1,12)_ = 3.343, *p* = 0.092). For theta, there was a significant decrease in power across sessions within a day (session *F*_(2,24)_ = 4.580, *p* = 0.021) but this did not differ between genotypes (session x genotype interaction *F*_(2,24)_ = 0.337, *p* = 0.717) or across days (session x day interaction *F*_(2,24)_ = 1.128, *p* = 0.340). Neither slow nor medium gamma power differed across sessions within each day (slow gamma: session *F*_(2,24)_ = 0.204, *p* = 0.817); medium gamma: session *F*_(2,24)_ = 0.184, *p* = 0.265). In order to examine whether our findings are dependent on rigid definition of frequency bands, we analysed the power spectrograms across the rats of each genotype for each session (Additional file 1: Fig. S9B). Our analyses show no significant differences between genotypes at any frequency range during any session.

As oscillatory power is affected by speed of movement [71, 72], we also compared the power of theta, slow gamma and medium gamma oscillations between genotypes at different speeds (Additional file 1: Fig. S10). However, these additional analyses did not reveal any significant genotype x velocity interactions (*p*’s > 0.05), indicating that in this dataset, hippocampal oscillatory power in the frequency ranges we analysed did not differ significantly between genotypes at any velocity.

### Pyramidal neurons from *Fmr1*^−/*y*^ rats exhibit decreased discharge modulation by gamma oscillations

**Fig. 8:**
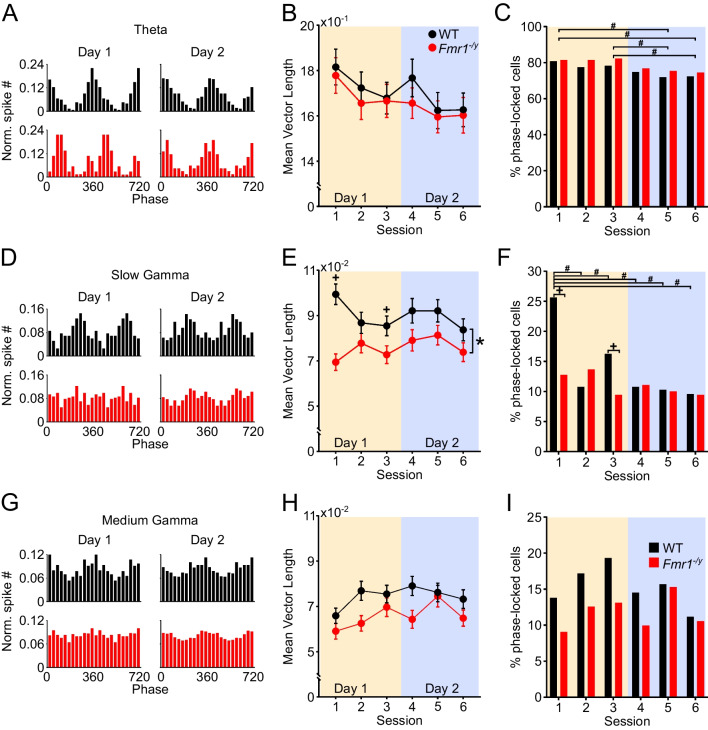
*Fmr1*^−/*y*^ CA1 pyramidal neurons are less phase locked to gamma oscillations than WT neurons. **A** Representative firing phase distribution of a single principal neuron along a theta cycle. Same for slow gamma oscillations (**D**) and medium gamma oscillations (**G**). **B** Average Mean Vector Length (MVL), quantifying the strength of phase locking to theta oscillations across the six recording sessions. MVL decreased significantly in cells of both genotypes across sessions within a day (session-in-day effect *p* = 0.026), but there were no differences between genotypes or days (*p*’s > 0.05). **C** The proportion of significantly phase-locked (Rayleigh *p* < 0.05) pyramidal neurons to theta oscillations across six recording sessions. There were no differences between genotypes, and no genotype x session interaction (*p*’s > 0.05). However, there is a significant main effect of session (Log-likelihood ratio: session effect *p* = 0.029), with theta phase locking higher in sessions 1 and 3 compared to sessions 5 and 6 (two proportion z-test, session 1 vs session 5 ^**#**^*p* < 0.01; session 1 vs session 6 ^**#**^*p* < 0.01; session 2 vs session 5 ^**#**^*p* < 0.05; session 2 vs session 6 ^**#**^*p* < 0.01) **E** WT neurons exhibit higher MVLs (stronger phase locking) to slow gamma compared to *Fmr1*^−/*y*^ neurons (LME: main effect of genotype *p = 0.022), with no significant differences between days or sessions within a day (*p*’s > 0.05). Further tests indicate significant differences between genotypes only in sessions 1 and 3 (Tukey *post hoc,* session 1 ^+^*p* < 0.001; session 3 ^+^*p* = 0.02). **F** A higher proportion of WT pyramidal neurons is significantly phase locked to slow gamma oscillations compared to *Fmr1*^−/*y*^ neurons (Log-likelihood ratio: genotype effect *p* = 0.035) and sessions different between one another (Log-likelihood ratio: session effect *p* < 0.001). While there was no significant genotype x session interaction (Log-likelihood ratio: genotype x session *p* = 0.053), the proportion of slow gamma phase locked neurons was higher in WT that rats in Sessions 1 and 3 of Day 1, but not for any other session (two proportion z-test, WT vs *Fmr1*^−/*y*^ Session 1: ^+^*p* < 0.001; Session 3: ^+^*p* = 0.023) a significantly higher proportion of WT neurons were phase-locked in session 1 than any other session (two proportion z-test WT, session 1 vs session 2 ^**#**^*p* < 0.001; session 1 vs session 3 ^**#**^*p* = 0.014; session 1 vs session 4 ^**#**^*p* < 0.001; session 1 vs session 5 ^**#**^*p* < 0.001; session 1 vs session 6 ^**#**^*p* < 0.001). **H** There were no significant differences in MVL with respect to medium gamma between genotypes, days or sessions within a day (LME all *p*’s > 0.05). **I** However, a significantly higher proportion of WT than *Fmr1*^−/*y*^ neurons was significantly phase locked to medium gamma oscillations (Log-likelihood ratio: genotype effect *p* = 0.013). *N*_WT-D1_ = 222, *N*_WT-D2_ = 207, *N*_KO-D1_ = 211, *N*_KO-D2_ = 205. Pale yellow and pale purple backgrounds denote data from Day 1 and Day 2, respectively

To examine the temporal organization of CA1 pyramidal neuron firing relative to theta, slow gamma and medium gamma oscillations, we computed the mean vector length (MVL) of the phase distribution of spikes for each pyramidal neuron for each of these frequency bands. This provides a measure of how consistently a neuron fires at a specific phase of the oscillation. We first compared the MVLs of all recorded CA1 pyramidal neurons in *Fmr1*^−/*y*^ and WT rats across sessions and days (LME analysis with genotype, day and session-in-day as fixed factors, animal and cell as random factors). The MVL for theta (Fig. 8A, B) did not differ between genotypes or between days (LME: genotype effect *p* > 0.05, day effect *p* > 0.05). However, there was a significant decrease in MVL across sessions within a day (session-in-day effect *p* = 0.026), with no significant interactions. In contrast, neurons from *Fmr1*^−/*y*^ rats showed significantly less phase locking (shorter MVLs) to slow gamma than those from WT rats (Fig. 8D, E; LME genotype effect *p* = 0.023), with no differences between days (LME day effect *p* = 0.537) or sessions within a day (LME session-in-day *p* = 0.057) and no significant interactions (LME day x genotype *p* = 0.109). Despite the absence of a significant day x genotype interaction, inspection of the data indicated that the difference between genotypes was larger on Session 1 of Day 1 than in later sessions. Thus, the significant main effect of genotype may have been driven primarily by the Session 1 differences in MVL. To address this possibility, we analysed the data for Day 1 and Day 2 separately. This revealed a significant genotype x session interaction on Day 1 (LME genotype x session *p* = 0.011), with *post hoc* tests indicating significant differences between genotypes on sessions 1 and 3 (*p* < 0.05). On Day 2, there was no significant difference between genotypes (LME genotype effect *p* = 0.374) and no genotype x session interaction (LME genotype x session interaction *p* = 0.852). An interpretation of this analysis is that the *Fmr1*^−/*y*^ rats show less phase locking to slow gamma on Day 1, but not on Day 2. For medium gamma (Fig. 8G, H), there were no significant differences in MVL between genotypes (LME *p* = 0.136), or days (LME *p* = 0.082), but there was a significant difference between sessions within a day (LME session in day *p* = 0.046), with no significant interactions.

To test whether the observed differences in phase locking of pyramidal cells to slow gamma between WT and of *Fmr1*^−/*y*^ rats may be secondary to differences in mean firing rate, we conducted a similar bootstrapping-like downsampling procedure as described previously for spatial metrics and firing rate map correlations. This analysis indicated that the mean firing rate differences between WT and *Fmr1*^−/*y*^ cells on Day 2 cannot explain the genotypic differences in phase locking to slow gamma oscillations (Additional file 1: Figure S12; Additional file 2: Table S8).

To further probe the modulation of CA1 spiking activity by oscillatory activity, and to allow direct comparison to previous findings in mice, we next quantified the proportion of recorded CA1 pyramidal neurons that were *significantly* phase locked to theta, slow gamma and medium gamma oscillations (MVL Rayleigh *p* < 0.05). Consistent with the previous analysis, the proportion of neurons (across all rats of each genotype) that were significantly phase locked to theta did not differ significantly between genotypes. However, there was a significant effect of session (Fig. 8C; Log-likelihood ratio: genotype x session *G*^2^ = 0.552, *p* = 0.990; genotype *G*^2^ = 2.96, *p* = 0.129; session *G*^2^ = 12.50, *p* = 0.029; for details of analysis see methods). Additional analyses revealed that theta phase locking was higher in sessions 1 and 3 compared to sessions 5 and 6, for both WT and *Fmr1*^−/*y*^ rats (two proportion z-test, session 1 vs session 5 *p* < 0.01; session 1 vs session 6 *p* < 0.01; session 2 vs session 5 *p* < 0.05; session 2 vs session 6 *p* < 0.01).

For slow gamma (Fig. 8F), the proportion of neurons that were significantly phase locked differed significantly between genotypes and across sessions (Log-likelihood ratio: genotype x session *G*^2^ = 10.92, *p* = 0.053; genotype *G*^2^ = 4.459, *p* = 0.035; session *G*^2^ = 21.19, *p* < 0.001). Given that the genotype x session interaction approached significance, we compared across sessions for each genotype separately. This revealed that the proportion of slow gamma phase locked neurons was higher in WT that rats in Sessions 1 and 3 of Day 1, but not for any other session (two proportion z-test, WT vs *Fmr1*^−/*y*^ Session 1: *p* < 0.001; Session 3: *p* = 0.023). Moreover, a significantly higher proportion of WT neurons were phase-locked in Session 1 than any other session, whereas there were no differences across sessions for *Fmr1*^−/*y*^ rats (two proportion z-test WT, session 1 vs session 2 *p* < 0.001; session 1 vs session 3 *p* = 0.014; session 1 vs session 4 *p* < 0.001; session 1 vs session 5 *p* < 0.001; session 1 vs session 6 *p* < 0.001; *Fmr1*^−/*y*^ all comparisons *p* > 0.05).

Finally, for medium gamma (Fig. 8I) there was a significant difference between genotypes in the proportion of pyramidal neurons that were phase-locked to medium gamma, but no significant interactions (Log-likelihood ratio: genotype x session *G*^2^ = 2.178, *p* = 0.824; genotype *G*^2^ = 6.24, *p* = 0.013; session *G*^2^ = 8.42, *p* = 0.135).

Collectively, these analyses reveal that the population of CA1 pyramidal neurons from *Fmr1*^−/*y*^ rats was less strongly phase locked to slow gamma oscillations compared to those of WT rats, particularly on Day 1. The overall strength of phase locking across the cell population to medium gamma did not differ significantly between WT and *Fmr1*^−/*y*^ rats, but fewer neurons from *Fmr1*^−/*y*^ rats were significantly phase locked to medium gamma. Thus, the timing of CA1 pyramidal neuron firing relative to both slow and medium gamma oscillations is less consistent in *Fmr1*^−/*y*^ compared to WT rats. In addition, in WT (but not in *Fmr1*^−/*y*^ rats) significantly more neurons were phase-locked to slow gamma in Session 1 on Day 1 (when the environment was completely novel) than in any other session. This provides further evidence of disruption in experience-dependent changes in CA1 neuronal activity in *Fmr1*^−/*y*^ rats. Individual cell data and rat average data are shown in Additional file 1: Figure S11 and LME analyses in Additional file 2: Table S7.

### Theta and gamma phase preferences of CA1 pyramidal neurons differ between WT and *Fmr1*^−/*y*^ rats

Another measure that can be used to characterize the organization of ensemble activity patterns is the timing of CA1 pyramidal neuron spiking relative to the theta and gamma oscillations. Inputs to the CA1 area of hippocampus have specific temporal organization, with inputs from CA3 arriving predominantly during the descending phase of CA1 theta, and inputs from MEC3 neurons primarily during the ascending phase and peak of theta [73, 74] (Fig. 8A). Moreover, CA1 pyramidal neurons have been reported to change their preferred theta phase (to the ascending phase of the theta cycle) in response to novelty [75]. Less is known about the functional significance of CA1 pyramidal neuron firing during specific gamma phases, although a correlation between slow gamma phase preference of CA1 pyramidal neurons and the position of their place fields in a linear track has been reported [76]. Taking into account that *Fmr1*^−/*y*^ rats exhibit decreased phase modulation by slow gamma oscillations and the fact that inputs from CA3 (reflected in slow gamma power) arrive predominantly during the descending phase of theta, we predicted that *Fmr1*^−/*y*^ CA1 pyramidal neurons would show decreased firing preference for the descending phase of theta compared to WT neurons.

**Fig. 9:**
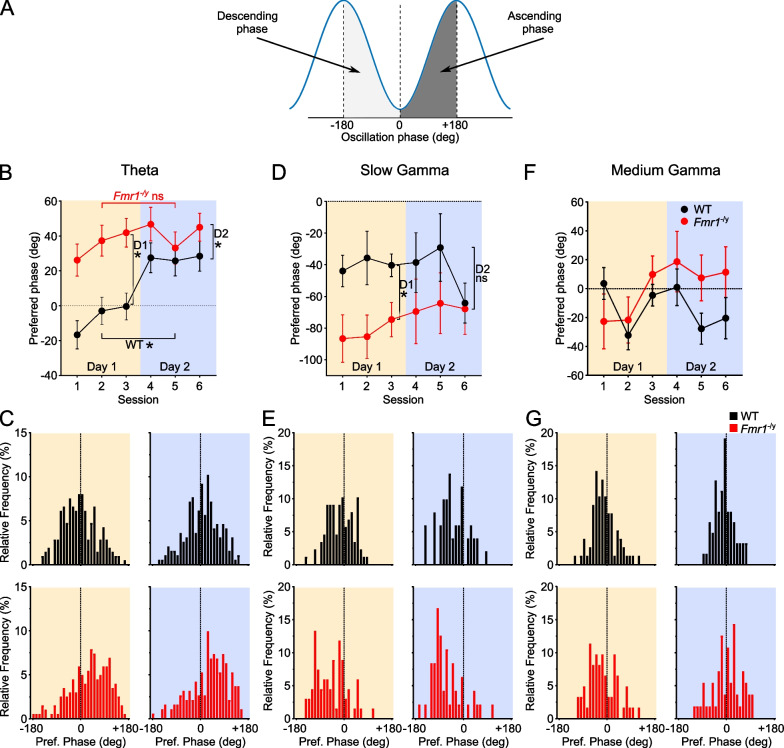
Theta and gamma phase preferences of CA1 pyramidal neurons differ between WT and *Fmr1*^−/*y*^ rats. **A** Schematic depiction of oscillation phases. The oscillation troughs were defined as 0°. **B** Mean preferred theta phase for significantly phase-locked (Rayleigh *p* < 0.05) CA1 pyramidal neurons during the six recording sessions. *Fmr1*^−/*y*^ neurons fired during the ascending phase of theta on both days, whereas WT neurons fired significantly earlier (at the trough of theta) on Day 1, and shifted to the ascending phase of theta on Day 2 (Harrison-Kanji test: main effect of genotype *p* < 0.001; genotype x day interaction *p* = 0.0025; Watson-Williams *post hoc* tests Day 1 vs Day 2: WT *p* < 0.001, *Fmr1*^−/*y*^* p* > 0.05; WT vs *Fmr1*^−/*y*^: Day 1 *p < 0.001, Day 2 *p < 0.001). **C** Distribution of preferred theta phase for unique pyramidal neurons recorded from WT (Top-black) and *Fmr1*^−/*y*^ (Bottom-red) rats during Day 1 (left) and Day 2 (right). **D** Same as (**B**) for slow gamma phase. *Fmr1*^−/*y*^ pyramidal neurons fired earlier during the descending phase of the oscillation compared to WT (Harrison-Kanji test: main effect of genotype: *p* = 0.003) (Watson-Williams *post hoc* tests*,* Day 1 *p < 0.05; Day 2 *p* = 0.103). This effect was driven by differences between genotypes during Day 1 (). **E** Same as (**C**) for slow gamma oscillations. **F** Same as (B) for medium gamma phase. No differences between genotypes in the preferred medium gamma phase (*p* > 0.05). **G** Same as (**C**) for medium gamma oscillations. Theta: *N*_WT-D1_ = 214, *N*_WT-D2_ = 197, *N*_KO-D1_ = 199, *N*_KO-D2_ = 187, SGamma: *N*_WT-D1_ = 89, *N*_WT-D2_ = 51, *N*_KO-D1_ = 65, *N*_KO-D2_ = 47, SGamma: *N*_WT-D1_ = 78, *N*_WT-D2_ = 63, *N*_KO-D1_ = 60, *N*_KO-D2_ = 56. Pale yellow and pale purple backgrounds denote data from Day 1 and Day 2, respectively

We analysed the preferred theta, slow gamma and medium gamma phases of CA1 pyramidal neurons from *Fmr1*^−/*y*^ and WT rats. Only neurons with significant phase modulation (based on MVL Rayleigh *p* < 0.05) were included in our analysis. Due to the circular nature of the data, we used the Harrison-Kanji two-factor test for circular data and the Watson-Williams multi-sample test to explore differences between genotypes and days. As can be seen in Fig. 9B, C, WT pyramidal neurons fired preferentially around the trough of theta during the first day of exposure to the novel environment, but more towards the ascending phase on the second day. In *Fmr1*^−/*y*^ rats, pyramidal neurons fired predominantly during the ascending phase of theta on both Day 1 and Day 2 (Harrison-Kanji test, main effect of genotype *p* < 0.001; genotype x day interaction *p* = 0.0025; Watson-Williams tests, Day1 vs Day2: WT *p* < 0.001; *Fmr1*^−/*y*^* p* = 0.858; WT vs *Fmr1*^−/*y*^: Day1 *p* < 0.001; Day2 *p* < 0.001). Analysis of the slow gamma preferred spiking phase (Fig. 9D, E) revealed that both WT and *Fmr1*^−/*y*^ pyramidal neurons fired primarily during the descending phase of the oscillation (negative preferred phases), but that *Fmr1*^−/*y*^ neurons fired at an earlier phase of slow gamma than WT neurons (Harrison-Kanji test, main effect of genotype: *p* = 0.003), with no difference in phase between Day 1 and Day 2 (main effect of day: *p* = 0.614), and no significant genotype x day interaction (*p* = 0.664). *Post hoc* tests exploring the main effect of genotype further revealed a significant difference in slow gamma phase preference between WT and *Fmr1*^−/*y*^ cells on Day 1 (*p* < 0.05) but not on day 2 (*p* = 0.103). Finally, medium gamma phase preference was not significantly different between WT and *Fmr1*^−/*y*^ rats (Harrison-Kanji test, main effect of genotype: *p* = 0.055), with no phase differences between days (*p* = 0.134), and no genotype x day interaction (*p* = 0.419) (Fig. 9F, G).

To explore whether the differences in preferred firing phases relative to theta between WT and *Fmr1*^−/*y*^ cells were driven by genotypic differences in mean firing rate, we conducted a downsampling analysis as described previously. This showed that theta phase preference differences were not secondary to mean firing rate differences between genotypes. Given the low numbers of cells that were significantly phase locked to gamma oscillations, we did not perform a downsampling analysis for slow and medium gamma phase preference.

Together, these analyses indicate that *Fmr1*^−/*y*^ pyramidal neurons exhibit differences in their preferred phase discharge compared to their WT counterparts. For theta, this difference is more prominent during the exploration of a novel environment (Day 1), while for slow gamma, the difference persists throughout both days of recordings.

## Discussion

Individuals with FXS have intellectual disability and ASD, two features in which hippocampal pathophysiology has been implicated [18, 77, 78]. While much is known about the cellular physiology and biochemical changes associated with the loss of FMRP, less is known about how cellular pathology leads to circuit dysfunction. The recent findings in the mouse models of FXS [20–24, 79] and the conserved hippocampal pathophysiology between *Fmr1*^−/*y*^ mice and rats [18] led us to predict that aspects of temporal coordination of both spiking activity and LFP would be affected in *Fmr1*^−/*y*^ rats. We also predicted that experience-dependent features of hippocampal cellular activity and spatial coding would be affected in *Fmr1*^−/*y*^ rats, as we and others have previously shown alterations in hippocampal synaptic plasticity [9, 18, 20]. The current findings indicate that CA1 pyramidal neurons in *Fmr1*^−/*y*^ rats display significant impairments in adaptation to repeated presentations of a novel spatial environment. Specifically, while WT neurons exhibit experience-dependent changes in firing rate, bursting, spatial tuning and spike timing relative to theta oscillations across days in a novel environment, these changes are absent in *Fmr1*^−/*y*^ rats. This absence of neurophysiological changes with repeated experience in a novel environment is reminiscent of a core clinical feature of FXS patients; abnormal habituation to novelty [80, 81]. *Fmr1*^−/*y*^ rats also showed less phase locking to gamma oscillations in an experience-independent fashion. Our in vivo findings are supported by ex vivo recordings that provide evidence for single cell hyperexcitability in the face of reduced synaptic inputs from the medial entorhinal cortex. Using ex vivo electrophysiological recordings, we showed that the circuit function abnormalities we observe may be, at least in part, the result of reduced synaptic inputs from MEC3 to CA1. Together, these data provide novel insight into how the absence of FMRP can disrupt neural circuit function that may lead to cognitive difficulties.

### Hippocampal CA1 pyramidal neuronal activity does not exhibit an experience-dependent decrease in *Fmr1*^−/*y*^ rats

We found that the firing rate and burstiness of CA1 pyramidal neurons are modulated by experience in WT rats, as has been shown previously [29, 62, 82]*,* but that these changes were absent in *Fmr1*^−/*y*^ rats. The decrease in mean firing rate with experience in a novel environment is thought to be mediated by synaptic plasticity, whereby synaptic inputs to CA1 pyramidal neurons are weaker in a familiar environment than in a novel environment [60]. Strong synaptic inputs during exploration of the novel environment (Day 1) would therefore lead to higher spiking activity in WT CA1 pyramidal neurons compared to the second day of recording in the same environment, when inputs are weaker. Our ex vivo experiments suggest that *Fmr1*^−/*y*^ CA1 pyramidal neurons exhibit increased excitability to compensate for the diminished synaptic inputs from MEC3 (Fig. 5). We propose that this homeostatic response allows the CA1 neurons in *Fmr1*^−/*y*^ rats to show high levels of activity in the novel environment, similar to WT rats on Day 1. However, as the environment becomes familiar (lower MEC3 to CA1 inputs), the increased excitability of *Fmr1*^−/*y*^ CA1 pyramidal neurons leads to sustained increase in firing rate. We have previously shown a similar functional effect (i.e. increased CA1 pyramidal neuron excitability) in the dorsal hippocampus of a mouse model of FXS [12]. Interestingly, the mechanisms mediating increased CA1 excitability appears to differ between rats and mice (e.g. increased AIS length), suggesting that while this alteration to hippocampal circuit function in the *Fmr1*^−/*y*^ rodents may be species independent, the mechanism by which mice and rats compensate for such change is divergent.

Our data may provide some reconciliation of previous findings in *Fmr1*^−/*y*^ KO mouse models, which had reported inconsistent effects of FMRP loss on CA1 pyramidal cell firing rates. Specifically, Boone et al. [21] reported higher CA1 pyramidal neuron firing rates in *Fmr1*^−/*y*^ than in WT mice exploring a highly familiar environment. In contrast, no differences in mean firing rate were reported between genotypes in the study by Talbot et al. [20] in which mice were recorded in several different environments (familiarity of each environment not specifically stated). In the study that is most similar to ours, Arbab et al. [24] recorded from CA1 pyramidal cells of mice over two consecutive days in an initially novel environment (Day 1, two 30 min sessions with 2 h ISI; Day 2, one 30 min session). They also reported no differences in firing rate between genotypes in any session. Overall, we did not find significant firing rate differences between WT and *Fmr1*^−/*y*^ rats, which is consistent with the findings of both the Talbot and Arbab studies. Instead, we found that cells in *Fmr1*^−/*y*^ rats failed to show the experience-dependent decrease in firing rate observed in WT rats. This phenotype was likely not observed in the Arbab et al. study because unlike the WT rats in our study, the WT mice in the Arbab et al. study did not show any experience-dependent changes in firing rate between sessions either within or between days. This lack of experience-dependent changes in CA1 pyramidal cell firing rate in a novel environment in WT mice is consistent with a previous study in WT mice [28] and may reflect a species difference between rats and mice in terms of how CA1 pyramidal cellular activity changes with environmental experience, and/or the time frame or amount of experience over which it changes. Indeed, mice need a substantially longer period of familiarization and training to perform certain tasks compared to rats [83, 84]. The Boone et al. [21] result is consistent with this account, as they observe higher CA1 pyramidal cell firing rates in *Fmr1*^−/*y*^ than WT mice in a highly familiar environment, which we suggest may be explained by experience-dependent changes occurring over a more extended period in WT mice (which is lacking in *Fmr1*^−/*y*^ mice), leading to differences between genotypes emerging only after many days of experience in an environment.

In addition to the decrease in mean firing rate, WT pyramidal neurons showed decreased bursting probability on Day 2 compared to Day 1, consistent with firing rate correlating to burst probability [85]. This phenomenon was absent in *Fmr1*^−/*y*^ pyramidal neurons. Given that the likelihood of bursting depends largely on the intrinsic excitability of neurons [61, 86], the pattern in *Fmr1*^−/*y*^ neurons may also be explained by the homeostatic increase in the excitability of *Fmr1*^−/*y*^ CA1 pyramidal neurons (Fig. 5). Specifically, reduction of mAHP has been shown to cause increased bursting [87]. In our data, the increase in single cell excitability was unrelated to a change in the length of the axon initial segment, which differs from our previous findings in *Fmr1*^−/*y*^ mice [12]. The reduced mAHP we observed may explain the hyperexcitability in CA1 pyramidal cells from adult *Fmr1*^−/*y*^ rats, which is consistent with previous findings in the ventral hippocampus in mice [13].

### Refinement of spatial information coding in the CA1 region of the hippocampus is impaired in *Fmr1*^−/*y*^ rats

The spatial information conveyed by hippocampal pyramidal neurons has previously been shown to increase sharply between the first and second day of exposure to a novel environment, both in mice [28] and rats [64]. We find a similar experience-dependent increase in spatial information of CA1 pyramidal neurons in WT rats between the first two days of exploration of a novel environment, but this is absent in *Fmr1*^−/*y*^ rats. As outlined in the results section, it is very unlikely that this pattern of findings is secondary to the subtle differences in exploratory behaviour between genotypes, in part because the genotypes differed from one another on both days, whereas the cellular properties change in WT rats between days. Moreover, our down-sampling control analyses suggest that the differences in spatial tuning in WT rats across days are not simply a consequence of the experience-dependent changes in firing rate in WT rats (Additional file 1: Fig. S12). The differences between WT and *Fmr1*^−/*y*^ rats may be due, at least in part, to the decreased strength of synaptic inputs to CA1 from MEC3 that we observed in *Fmr1*^−/*y*^ rats in our ex vivo experiments, and which we have previously reported in the *Fmr1*^−/*y*^ mouse [12]. Lesions of MEC3 result in large reductions in the spatial information of CA1 pyramidal neurons in rats exploring a familiar environment (where control animals show high spatial information), whereas they have much more subtle effects on spatial information in a novel environment (in which spatial information is low in both lesioned and control rats) [88]. This indicates that in WT rats, MEC3 inputs to CA1 typically contribute to the experience-dependent refinement of the spatial coding, and that the failure of *Fmr1*^−/*y*^ pyramidal neurons to show an increase in spatial information upon familiarization to the novel environment across days may result from the reduced strength of these synaptic inputs.

Comparing the current findings to the previous mouse studies, Arbab et al. [24] reported some spatial tuning deficits in *Fmr1*^−/*y*^ mice (larger place fields, more active pixels, and lower spatial specificity) both on Day 1 and Day 2 in an initially novel environment, with no changes in these parameters across days in either group. They did not observe any differences between genotypes in spatial information. In contrast, neither Boone et al. [21] (in which mice were recorded in a highly familiar environment), nor Talbot et al. [20] who recorded in a variety of environments) reported any differences in spatial tuning between WT and *Fmr1*^−/*y*^ mice. Considering these data together, it is possible that spatial tuning deficits in *Fmr1*^−/*y*^ mice do not persist in more familiar environments. While we cannot account for the differences between our rat findings and the mouse findings in a novel environment, both our findings and those of Arbab indicate decreased spatial tuning in *Fmr1*^−/*y*^ animals, albeit with a different dependence on experience within the novel environment. Indeed, the WT mice in the Arbab et al. study [24] did not show any increase in spatial tuning across sessions in the novel environment, making direct comparison challenging. In future studies of the rat model, it would be interesting to record across both very familiar and novel environments, and to record for more days in the novel environment, to establish a time course for the observed differences in spatial tuning of CA1 place cells in both WT and *Fmr1*^−/*y*^ rats.

### Stability of spatial firing rate maps in the CA1 region of the hippocampus does not differ between WT and *Fmr1*^−/*y*^ rats

While spatial tuning showed the expected refinement between days 1 and 2 in WT rats, the within-day, between-session stability of firing rate maps of pyramidal neurons did not increase from Day 1 to Day 2 in either WT or *Fmr1*^−/*y*^ rats. For both groups, the within-day stability was higher than the between-day stability (session 3 vs session 4), suggesting some remapping across days in both groups. This degree of remapping between the first two days in an initially novel environment with a total of 30 min exposure on Day 1 is comparable with that recently reported in WT rats [89]. However, we cannot exclude the possibility that the lower firing rate map correlations observed between days compared to within days may be due in part to poorer tracking of cells across days than across sessions within a day.

Contrary to our predictions, the firing rate maps of *Fmr1*^−/*y*^ rats were as stable as those of WT rats, both within and between days. This is not consistent with the findings of Arbab et al. [24] who reported that CA1 neurons from *Fmr1*^−/*y*^ mice exhibited lower firing rate map stability both within (comparing two halves of the session) and between two 30 min exposures to a novel environment compared to WT mice [20]. Our down-sampling analysis showed that this discrepancy is not due to firing rate map stability being affected by firing rate changes across days in our study. It is therefore surprising that we did not observe any differences in firing rate map correlations between genotypes. A variety of factors, including species differences, differences in the daily session number, session duration and within-day inter-trial intervals could have affected firing rate map stability in the two studies.

Experience-dependent increases in spatial tuning of CA1 pyramidal neurons depend on the autophosphorylation of the α-Isoform of the Calcium/Calmodulin-Dependent Protein Kinase II (α-CaMKII) [28]. However, the stability of firing rate maps is independent of this plasticity mechanism. Given that FMRP directly regulates α-CaMKII mRNA translation [6], we suggest that constitutive loss of FMRP may disrupt the plasticity mechanisms that mediate experience-dependent changes in spatial specificity, while leaving the plasticity processes mediating firing rate map stability unaffected.

### Hippocampal LFP power is largely unaffected by the loss of FMRP

Our LFP recordings from the CA1 pyramidal neuron layer did not reveal any significant differences between genotypes, or any experience-dependent changes in the power of theta (6–12 Hz), low-range gamma (30–45 Hz) or mid-range gamma (55–100 Hz) band activity. Crucially, these results as not dependent on the definition of frequency boundaries we used. Whole spectrogram analyses for every session did not reveal any significant differences either. At face value, these findings contradict previous reports in *Fmr1*^−/*y*^ mice in which stronger theta [22] and slow gamma [21–23] have been reported. However, power spectra analyses often fail to reveal changes in rhythms that other analyses, such as LFP coherence across multiple channels, can detect. Measures of power are also very dependent on the precise location of the recording electrodes relative to the CA1 pyramidal cell layer [68]. Therefore, we urge caution in over-interpreting the current findings, which suggest no impact on theta or gamma power. Indeed, although not significant, we did observe a trend towards higher slow and medium gamma power in *Fmr1*^−/*y*^ rats, echoing the findings from a number of studies reporting that slow gamma power is stronger in the hippocampus of *Fmr1*^−/*y*^ mice [21–23]. Beyond the hippocampus, increased gamma-band power has been reported in the frontal and temporal cortex of *Fmr1*^−/*y*^ mice [90], the parietal and temporal cortex of *Fmr1*^−/*y*^ rats [91], and in individuals with FXS [92], which suggest that network-wide deficits in either gamma oscillation generation or maintenance are conserved between mammalian species.

Given our ex vivo findings that MEC inputs are diminished in the absence of FMRP, we might have predicted weaker medium gamma power compared to WT. However, the anatomical organization of inputs to the hippocampus leads to large variations in the power of network oscillations between locations within the hippocampus [49, 69, 93]. Therefore, future work should include multisite recordings within hippocampal sub-layers and in extra-hippocampal input areas to determine the precise effects of FMRP loss on hippocampal circuit function [23, 79], as well as to delineate the contribution of the different inputs to the hippocampus [49].

### Weaker gamma phase locking and disrupted experience-dependent changes in gamma phase locking in CA1 pyramidal neurons of *Fmr1*^−/*y*^ rats

The spikes of pyramidal neurons in *Fmr1*^−/*y*^ rats were less strongly phase locked to the cycles of gamma oscillations than those of WT rats. The most robust difference in phase locking between WT and *Fmr1*^−/*y*^ rats were seen in the slow gamma range on Day 1, although the proportion of neurons that were significantly phase locked to medium gamma also differed between genotypes. Furthermore, the preferred firing phase of *Fmr1*^−/*y*^ neurons was earlier during the descending phase of slow gamma compared to WT pyramidal neurons. These results are consistent with a previous study in *Fmr1*^−/*y*^ mice, which reported weaker modulation of pyramidal neuron firing by slow gamma, unstable ensemble coordination in relation to slow gamma (40 Hz), and discharge preference for earlier slow gamma phases [24]. Less spiking modulation by slow gamma in *Fmr1*^−/*y*^ than WT rats suggests not only weaker coupling with extrinsic inputs (e.g. CA3, MEC), but may also reflect local microcircuit dysfunction, as has been shown in the developing neocortex [20, 94–96].

### Alterations in timing of CA1 pyramidal neuron firing relative to theta oscillations

WT neurons fired preferentially during the late descending phase of theta in the first session in the novel environment, and their activity moved towards the early ascending phase with repeated exposures across the two days. In contrast, *Fmr1*^−/*y*^ pyramidal neurons fired preferentially in the early ascending phase of theta on both days and showed no experience-dependent changes. Previous reports suggest that theta phase preference shifts towards the ascending phase during exploration of a novel environment [68, 75, 97]. Our results from WT rats directly contradict these findings. This inconsistency may be due to differences in the experimental protocol. It is plausible that the differences between the screening box and the novel environment used on Day 1 are too big (see Methods) and that leads to rats spending less time attending to or encoding the new spatial information (therefore no shift to ascending theta phase when inputs from MEC3 are the strongest) than expected. On Day 2 the rats may be more attentive to environment features and that is when we observe the shift. Moreover, in previous studies, theta phase preference was compared between a novel and a very familiar environment, whereas our study did not include recording in a very familiar environment, which precludes a direct comparison.

Interestingly, the preference of *Fmr1*^−/*y*^ pyramidal neurons to fire during the ascending phase of theta is nearly identical with a previous study in *Fmr1*^−/*y*^ mice [20], and may suggest abnormal synaptic drive, arising from either excitatory or inhibitory inputs. Our ex vivo data revealed that MEC3 synaptic strength is decreased in *Fmr1*^−/*y*^ rats. Given the known theta phase segregation of inputs to CA1 (MEC3 inputs strongest at the ascending phase and peak of the theta; [74]), based on this finding alone, we might have predicted decreased CA1 firing during the ascending phase of theta in *Fmr1*^−/*y*^ rats. However, the ex vivo recordings also revealed that altered intrinsic excitability is able to compensate (in part) for spike output, which is consistent with our previous findings in *Fmr1*^−/*y*^ mice [12]. Together, this suggests that reduced excitatory drive is compensated for within the local circuit to normalize CA1 population activity in *Fmr1*^−/*y*^ rats.

Given the precise temporal organization of inhibition in the hippocampus [98, 99] and the known abnormalities in hippocampal inhibitory transmission in the absence of FMRP [100], it is tempting to speculate that the absence of experience-dependent change in the theta phase preference observed in *Fmr1*^−/*y*^ pyramidal neurons is, at least in part, due to a persistent decrease of inhibitory tone during the ascending phase of theta in *Fmr1*^−/*y*^ rats, which permits pyramidal neuron discharge during that phase. It would be very interesting to explore the organization of hippocampal activity by theta oscillations further. Using multisite probes that allow better monitoring of neural activity across hippocampal subfield, and allowing animals to explore environments with narrow alleys, phenomena such as theta phase precession can be studied more easily [101]. Interestingly, a mouse model of a different neurodevelopmental disorder (Down syndrome) has been shown to exhibit abnormal theta phase precession in the absence of spatial tuning abnormalities in hippocampal place cells [102].

### Implications for behavioural deficits associated with loss of FMRP

Overall, *Fmr1*^−/*y*^ rats in the current study did not display the experience-dependent changes in firing and spatial tuning of CA1 place cell activity seen in WT littermates. The failure to update their spatial coding of a novel environment across days may reflect an inability of *Fmr1*^−/*y*^ rats to discriminate subtle spatial and contextual information. In contrast, firing rate map stability between sessions and days does not differ between genotypes. This suggests that, to the extent that firing rate maps reflect recognition of the environment, *Fmr1*^−/*y*^ rats would not be impaired in their ability to recognize that they are in a familiar environment. Our limited behavioural measures (pathlength) in the current study also support this conclusion, as there is a decrease in pathlength both within each day and between days (and it is known that rats tend to explore novel more than familiar environments). However, further behavioural studies are required to establish whether indeed the *Fmr1*^−/*y*^ rats can detect the novelty/familiarity of the environment. This could be done by measuring additional exploratory behaviours known to be associated with environmental novelty, such as rearing, across repeated exposures to a novel environment across days (in a situation in which exploration is not being motivated by scattered treats in the environment), or by testing the animals in a task in which they are required to make different responses for rewards in novel vs familiar environments.

Increased phase locking of CA1 pyramidal neurons to slow gamma is believed to be involved in memory retrieval processes [23, 76, 103]. Therefore, taking into account the reduced locking of *Fmr1*^−/*y*^ CA1 pyramidal neurons to slow gamma, it is plausible that fine spatial information retrieval is affected in *Fmr1*^−/*y*^ rats, and that underlies their inability to increase their spatial tuning over days. Indeed, previous work has shown that *Fmr1*^−/*y*^ mice exhibit behavioural inflexibility when required to learn new spatial rules that conflict with previous knowledge and that this correlates with abnormally frequent events of strong slow gamma oscillations [23].

### Limitations

Our recordings span only two days of habituation to an initially novel environment. It is unclear whether, given enough experience, *Fmr1*^−/*y*^ CA1 pyramidal neurons would improve their spatial tuning and reduce their mean firing rates to levels comparable to WT littermates. Future work should explore habituation to novelty over longer time scales and increased experience. In addition, comparing activity in a novel environment with that in a very familiar environment would allow more direct comparisons with previous studies.

A second limitation is that our ability to track the same cells over days may not be as robust as the ability to track cells within a day. To minimize this possibility, spike sorting was conducted on the data from all 6 sessions over the two days combined, and all clusters were visually inspected to ensure that waveforms from a given cluster did not differ markedly from session to session or across days. Nonetheless, it is possible that there was more cluster drift between days than within days. Importantly, there is no reason why poor tracking of cells would differentially affect the data obtained from WT vs *Fmr1*^−/*y*^ rats. Moreover, none of the measures used to assess firing properties, spatial coding or spike timing within each session depended on having data from the same cells across sessions or days, as only data from “active” cells in a given session were included in these analyses, so they are effectively different (highly overlapping) populations of cells. The only exception to this was in the calculation of spatial firing rate map correlations, where the firing rate maps of individual cells were compared across sessions and days. It is therefore possible that the weaker correlations between days than within days may reflect poorer tracking of cells between days. As there were no differences in firing rate map correlations between genotypes, this limitation does not affect the conclusions of the current study. However, future experiments aimed at assessing stability of spatial firing over multiple days may be better served by using imaging techniques that allow definitive tracking of the same cells across days [104].

A third limitation relates to the observation that while WT cells showed significant decreases in activity and significant increases in spatial coding across days which were not observed in *Fmr1*^−/*y*^ cells, the groups did not differ significantly from one another on either day for these measures. While it is clear from the pattern of the data that the groups did not differ on Day 1, it is possible that the failure to observe significant differences between the groups on Day 2 may have been due in part to being underpowered to detect such a difference. For this reason, we have not drawn any conclusions based on whether or not the groups differed on Day 2, but rather, restricted our discussion to the lack of experience-dependent changes in the *Fmr1*^−/*y*^ cells.

Finally, all of our CA1 recordings were made from neurons approximately midway along the proximal–distal axis between CA3 and subiculum. Given the known spatial tuning gradient along the CA1 proximo-distal axis hippocampus [105], we should be cautious when extrapolating from our findings to spatial coding across the whole hippocampus.

## Conclusions

Overall, our findings offer new insights into circuit pathophysiology associated with the loss of FMRP and how that may lead to abnormal habituation to novelty. It would be interesting to see if hippocampal circuit deficits converge with those seen in other rodent models of ASD/ID. Interestingly, absence of experience-dependent update in spatial tuning has been reported previously in a mouse model of Rett syndrome [63], while reduced spatial specificity has also been observed in two different mouse models of Down syndrome [99, 106]. This raises the distinct possibility of conserved neuropathophysiology underlying cognitive abnormalities in ASD/ID models [107] and highlights that exploring the in vivo physiology of neurons in freely moving animals is critical to determining the functional consequences of neurodevelopmental disorders.

## Supplementary Information

**Additional file 1**. Table S1, Table S2, Fig. S1, Fig. S2, Fig. S3, Fig. S4, Fig. S5, Fig. S6, Fig. S7, Fig. S8, Fig. S9, Fig. S10, Fig. S11 and Fig. S12.

**Additional file 2**. Table S3, Table S4, Table S5, Table S6, Table S7, Table S8 and Table S9.

## Data Availability

The LE-*Fmr1*^*em1/PWC*^ rat line is available from SIDB under a material transfer agreement with the University of Edinburgh. All data and data analysis routines associated with this study are available upon request.
