# Supplementary material for: Experience-dependent changes in hippocampal spatial activity and hippocampal circuit function are disrupted in a rat model of Fragile X Syndrome
Source: Mol Autism. 2022 Dec 20;13:49. doi: 10.1186/s13229-022-00528-z (PMC9764562; doi:10.1186/s13229-022-00528-z)
Supplement: Supplementary file 1 — Additional file 1. Table S1, Table S2, Fig. S1, Fig. S2, Fig. S3, Fig. S4, Fig. S5, Fig. S6, Fig. S7, Fig. S8, Fig. S9, Fig. S10, Fig. S11 and Fig. S12. [file 13229_2022_528_MOESM1_ESM.pdf]

Supplementary Material

Asiminas et al. Experience-dependent changes in hippocampal spatial activity and hippocampal circuit function are disrupted in a rat model of Fragile X Syndrome

		Number of identified units in each session					
Rat	Genotype	1	2	3	4	5	6
1	KO	9	8	8	7	6	9
2	WT	23	21	21	25	26	26
3	KO	19	21	25	23	21	22
4	WT	16	17	14	15	15	17
5	WT	22	25	25	21	24	22
11	KO	61	56	57	52	47	49
12	KO	47	49	47	42	43	44
14	KO	33	31	36	42	35	39
15	KO	70	65	67	70	70	72
17	WT	47	41	40	49	48	50
18	WT	23	22	22	24	24	26
19	WT	36	37	36	35	33	31
20	WT	83	78	75	61	57	53
21	KO	13	13	13	4	4	4

Supplemental Table 1. Number of identified units for each rat in each recording session

	Percentage of units found present		
	Both Days	Only Day 1	Only Day 2
WT	51.59	26.8	21.61
KO	71.25	15.94	12.81

Supplemental Table 2. Percentage of cells identified throughout the experiment. ‘Both days’ includes percentages of WT and *Fmr1*$^{-/y}$ cells that were identified in at least one session in both days of the experiment. ‘Only Day 1’ and ‘Only Day 2’ shows the percentages of cells that were identified in at least one session of the first or the second day of the experiment respectively.

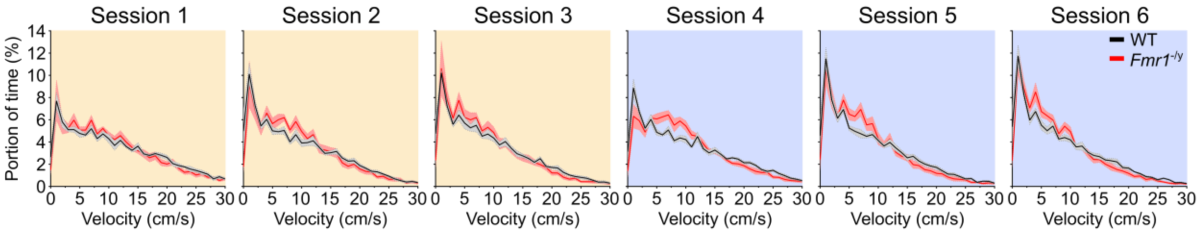

Figure S1. No differences in distribution of movement velocities between WT and *Fmr1*$^{-/y}$ rats. Movement velocity was calculated for every 500 ms epoch for each rat in each session. The figure depicts the proportion of time spent moving at different velocities from 0-30 cm/s (velocity bin width = 1s) across the six sessions for WT and *Fmr1*$^{-/y}$ rats. A 4-way mixed ANOVA (genotype, day, session-in-day, velocity bin) indicated no significant differences between genotypes (genotype $F_{(1,12)}$=2.211, p=0.163) and no significant interactions between genotype and any other variable (genotype x day $F_{(1,12)}$=0.006, p=0.938; genotype x session-in-day $F_{(2,24)}$=1.669, p=0.210; genotype x velocity $F_{(29,348)}$=1.432, p=0.073; genotype x day x session $F_{(2,24)}$=1.683, p=0.207; genotype x day x velocity $F_{(29.348)}$=0.654, p=0.917; genotype x session-in-day x velocity $F_{(58,696)}$=1.186, p=0.170; genotype xday x session-in-day x velocity $F_{(58,696)}$=0.889, p=0.707). Solid lines depict rat means; shaded areas depict SEM. Pale yellow and pale purple backgrounds denote data from Day 1 and Day 2 respectively.

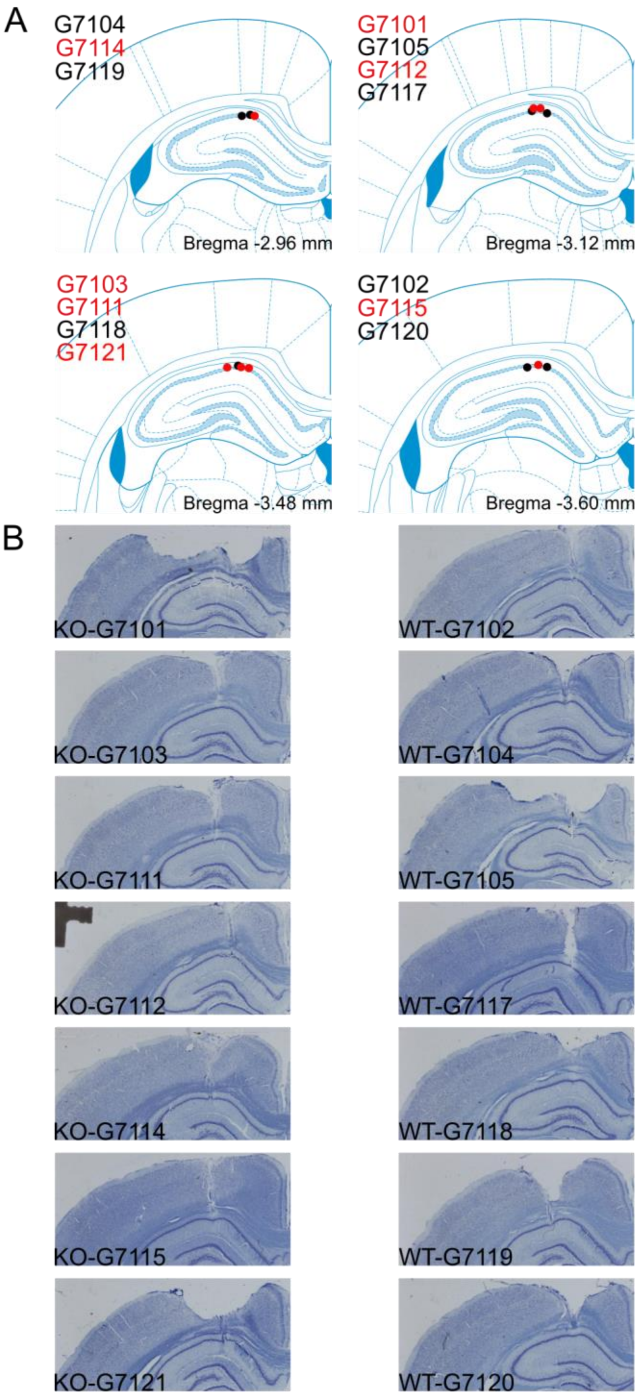

Figure S2. Histological confirmation of electrode placement. (A) Schematics of individual electrode placements in the CA1 cell layer of the hippocampus from all rats. Dots represent the location of the tip of the tetrode bundle at the end of recording. Each dot is labelled with a rat number and dot colour indicates genotype (black for WT and red for *Fmr1*$^{-/y}$). The four different schematics reflect the estimated anterior-posterior (AP) coordinate (AP -3.48 mm from bregma being the intended coordinate). **(B)** Coronal brain sections from all WT and an *Fmr1*$^{-/y}$ rat stained with cresyl violet.

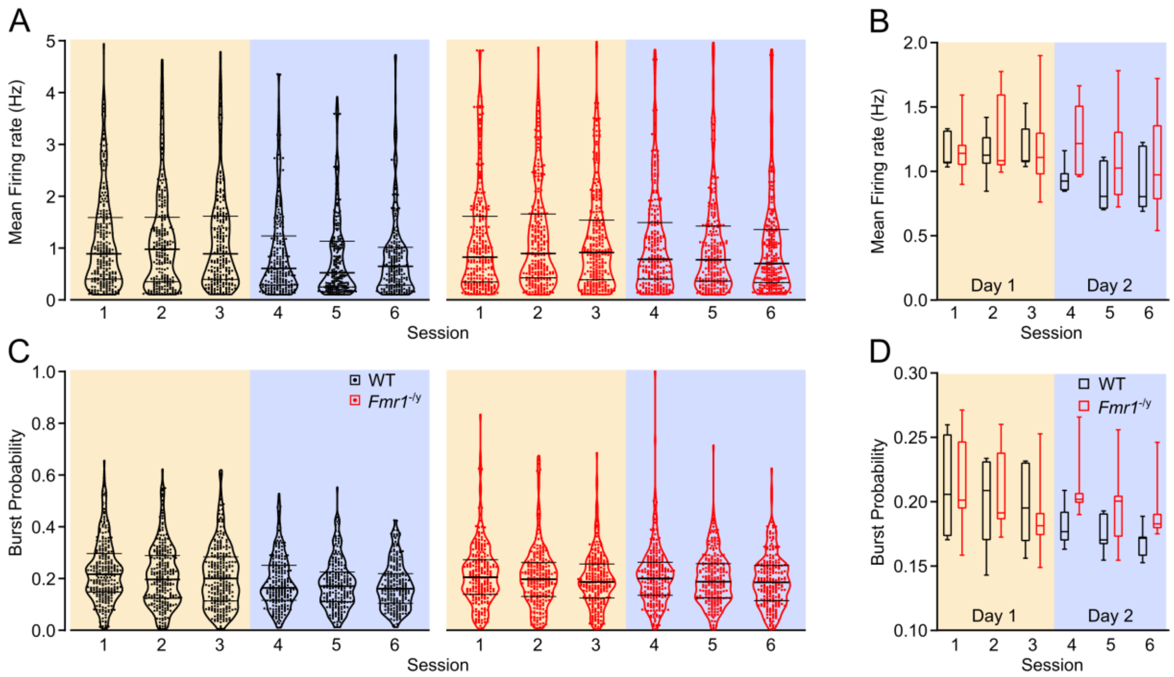

Figure S3. Overview of data distributions for each session and rat means level analysis for firing rate and burst probability (A) Violin plots depicting the mean firing rate distributions across all six recording sessions for WT and *Fmr1*$^{-/y}$ pyramidal neurons. **(B)** Mean firing rate plotted and analysed at the rat average level. Three-way RM ANOVA: Day x Session x genotype: $F_{(2,24)}$=0.788, p=0.466; Day x Genotype: $F_{(1,12)}$=1.949, p=0.188; Session x Genotype: $F_{(2,24)}$=1.089, p=0.353; Genotype: $F_{(1,12)}$=1.147, p=0.305; Day: $F_{(1,12)}$=5.933, p=0.031; Session: $F_{(2,24)}$=0.674, p=0.532. **(C-D)** Same as (**A-B**) for burst probability. Three-way RM ANOVA: Day x Session x genotype: $F_{(2,24)}$=0.427, p=0.657; Day x Genotype: $F_{(1,12)}$=7.005, p=0.021; Session x Genotype: $F_{(2,24)}$=0.246, p=0.784; Genotype: $F_{(1,12)}$=1.060, p=0.324; Day: $F_{(1,12)}$=6.165, p=0.029; Session: $F_{(2,24)}$=6.109, p=0.007. *Posthoc* tests Day1: WT vs *Fmr1*$^{-/y}$ p=0.974; Day2: WT vs *Fmr1*$^{-/y}$ p=0.041; WT Day1 vs Day2 p=0.034; *Fmr1*$^{-/y}$ Day1 vs Day2 p=0.820. For box and violin plots the middle line represents rat median, upper and lower end of the boxes, and upper and lower line in the violins represent 95th and 5th percentile, error bars in box plots represent maximum and minimum values. N_{WT} = 7, N_{KO} =7. Pale yellow and pale purple backgrounds denote data from Day 1 and Day 2 respectively.

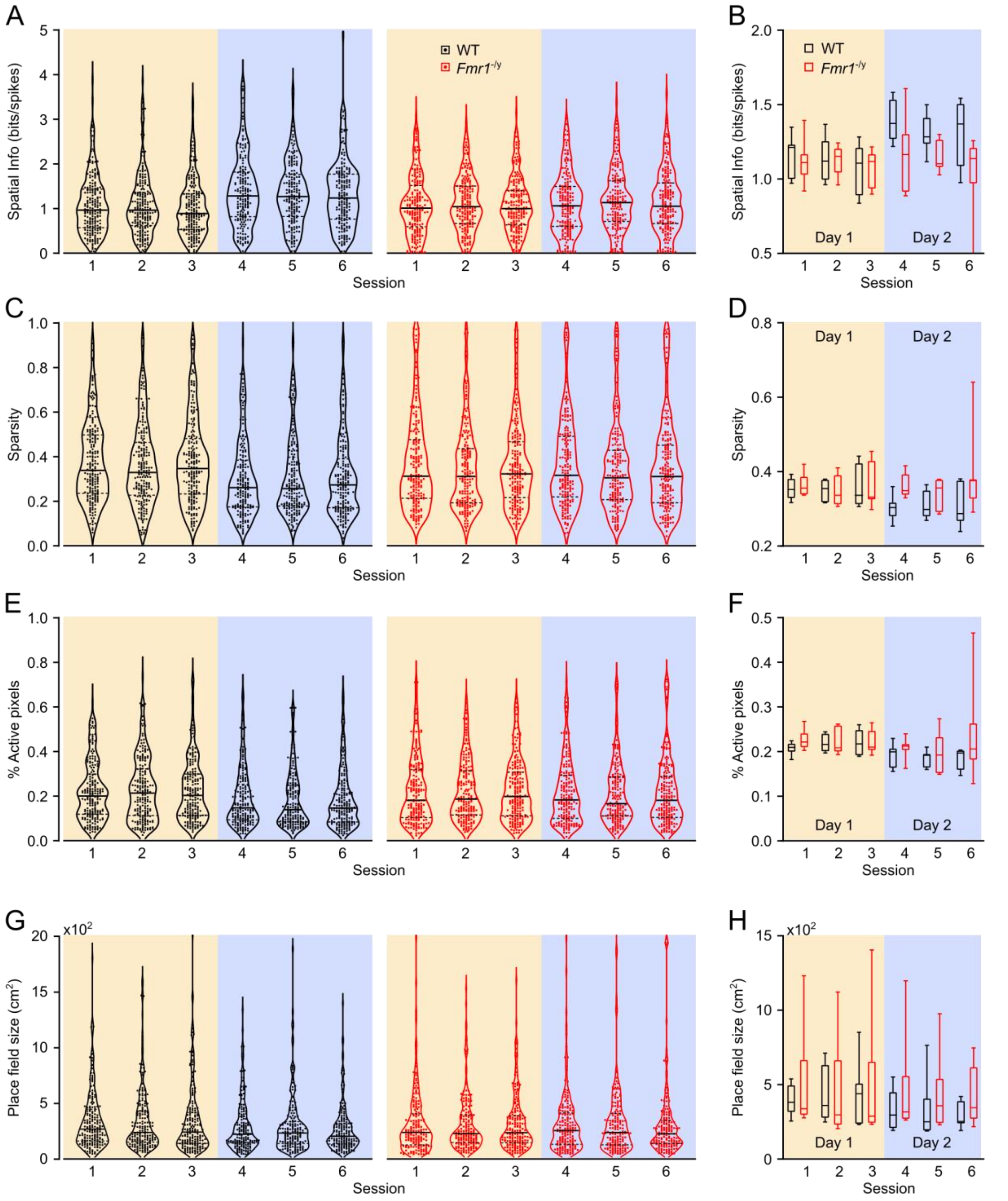

Figure S4. Overview of data distributions for each session and rat means level analysis for spatial firing metrics (A) Violin plots depicting the spatial information distributions across all six recording sessions for WT and *Fmr1*$^{-/y}$ pyramidal neurons. **(B)** Spatial information plotted and analysed at the rat average level. Three-way RM ANOVA: Day x Session x genotype: $F_{(2,24)}$=0.561, p=0.578; Day x Genotype: $F_{(1,12)}$=7.363, p=0.019; Session x Genotype: $F_{(2,24)}$=0.211, p=0.811; Genotype: $F_{(1,12)}$=2.875, p=0.116; Day: $F_{(1,12)}$=9.285, p=0.010; Session: $F_{(2,24)}$=2.132, p=0.141. *Posthoc* tests Day1: WT vs *Fmr1*$^{-/y}$ p=0.992; Day2: WT vs *Fmr1*$^{-/y}$ p=0.023; WT Day1 vs Day2 p=0.002; *Fmr1*$^{-/y}$ Day1

vs Day2 p=0.846. **(C-D)** Same as (**A**-**B**) for sparsity. Three-way RM ANOVA: Day x Session x genotype: $F_{(2,24)}$=0.762, p=0.478; Day x Genotype: $F_{(1,12)}$=8.472, p=0.013; Session x Genotype: $F_{(2,24)}$=0.687, p=0.513; Genotype: $F_{(1,12)}$=2.460, p=0.143; Day: $F_{(1,12)}$=5.189, p=0.042; Session: $F_{(2,24)}$=1.364, p=0.275. *Posthoc* tests Day1: WT vs *Fmr1*$^{-/y}$ p=0.948; Day2: WT vs *Fmr1*$^{-/y}$ p=0.018; WT Day1 vs Day2 p<0.001; *Fmr1*$^{-/y}$ Day1 vs Day2 p=0.745. **(E-F)** Same as (**A**-**B**) for %Active pixels. Three-way RM ANOVA: Day x Session x genotype: $F_{(2,24)}$=0.719, p=0.497; Day x Genotype: $F_{(1,12)}$=2.103, p=0.173; Session x Genotype: $F_{(2,24)}$=0.691, p=0.511; Genotype: $F_{(1,12)}$=2.560, p=0.136; Day: $F_{(1,12)}$=6.977, p=0.022; Session: $F_{(2,24)}$=0.687, p=0.513. **(G-H)** Same as (**A**-**B**) for Place field size. Three-way RM ANOVA: Day x Session x genotype: $F_{(2,24)}$=0.202, p=0.818; Day x Genotype: $F_{(1,12)}$=1.199, p=0.281; Session x Genotype: $F_{(2,24)}$=0.081, p=0.923; Genotype: $F_{(1,12)}$=1.628, p=0.210; Day: $F_{(1,12)}$=5.45, p=0.025; Session: $F_{(2,24)}$=0.016, p=0.984. For box and violin plots the middle line represents rat median, upper and lower end of the boxes, and upper and lower line in the violins represent 95th and 5th percentile, error bars in box plots represent maximum and minimum values. N_{WT} = 7, N_{KO} =7. Pale yellow and pale purple backgrounds denote data from Day 1 and Day 2 respectively.

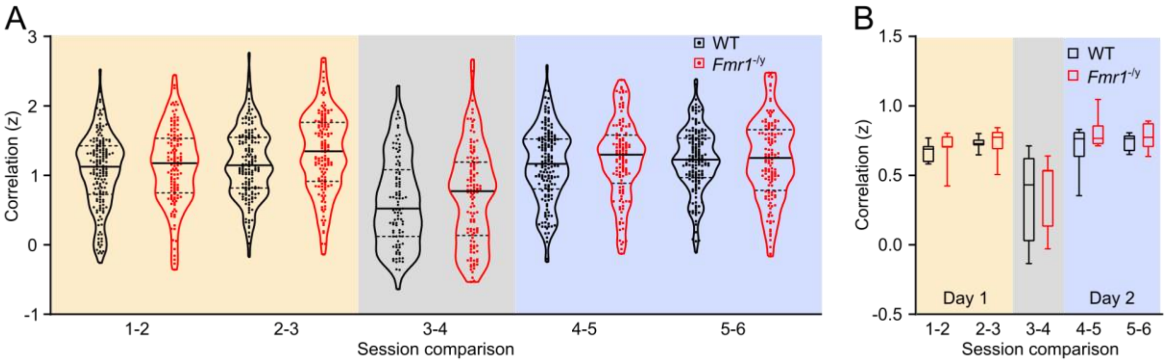

Figure S5 Overview of data distributions for each session and rat means level analysis for spatial stability (A) Violin plots depicting the Fisher z-transformed firing rate map correlation distributions across all five session comparisons for WT and *Fmr1*$^{-/y}$ place cells. **(B)** Firing rate map correlation plotted and analysed at the rat average level. Two-way RM ANOVA: Genotype x session comparison: $F_{(4,12)}$=0.143, p=0.965; Genotype: $F_{(1,12)}$=2.828, p=0.118; Session comparison: $F_{(4,12)}$=16.925, p<0.001. For box and violin plots in (**B**), the middle line represents rat median, upper and lower end of the boxes, and upper and lower line in the violins represent 95th and 5th percentile, error bars in box plots represent maximum and minimum values. N_{WT} = 7, N_{KO} =7. Pale yellow and pale purple backgrounds denote data from Day 1 and Day 2 respectively, while grey in (B) denotes comparison between the last session on Day 2 (Session 3) and the first session on Day 2 (Session 4).

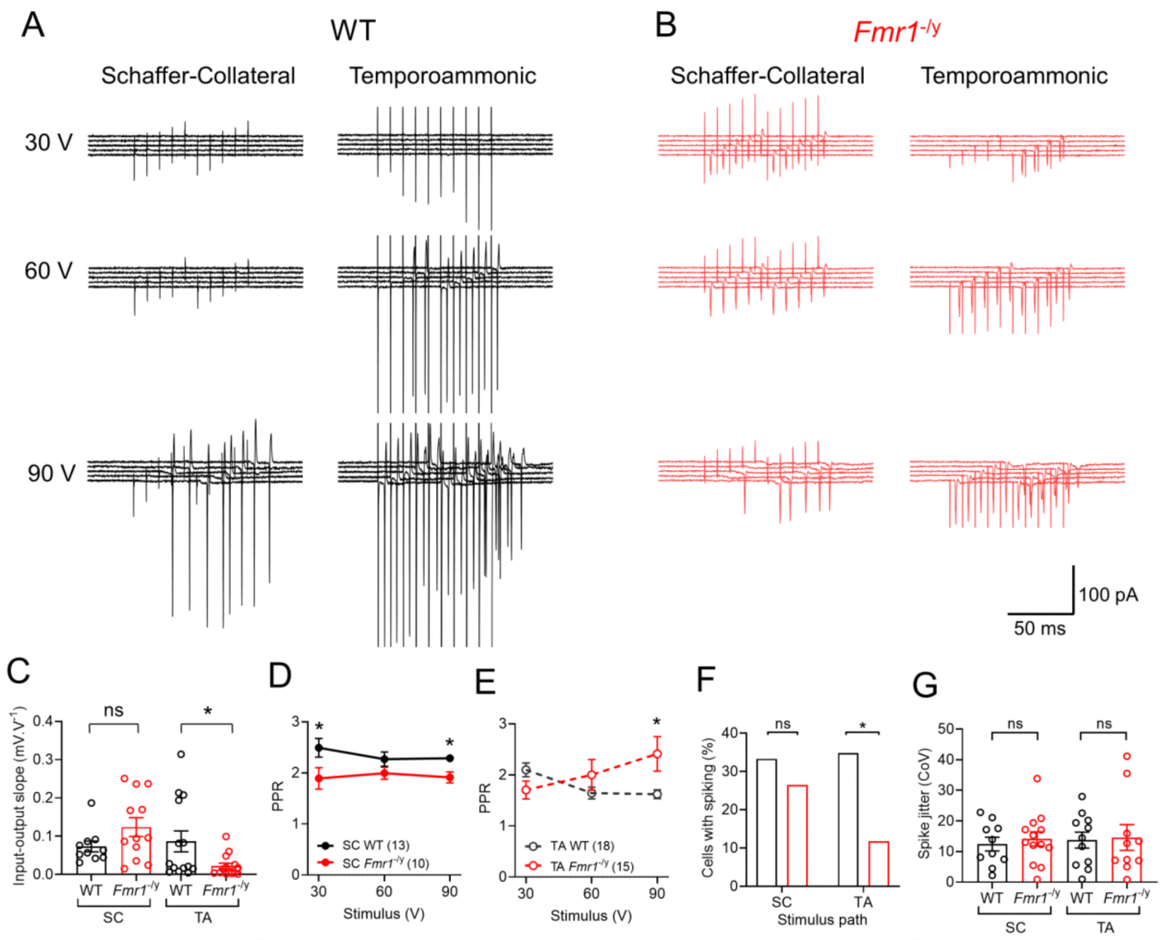

Figure S6. Genotype specific differences in synaptic and cellular recruitment at Schaffer-Collateral and Temporoammonic pathways. (A) Representative traces from a WT CA1 pyramidal neuron recorded in cell-attached configuration at the soma. 5 traces are shown at 30 V, 60 V, and 90 V stimulation delivered to the Schaffer-Collateral (left) or temporoammonic (right) paths in *str. radiatum* or *str. lacunosum-moleculare* respectively. **(B)** Representative traces performed under the same conditions as A, but for a pyramidal neuron from an *Fmr1*$^{-/y}$ rat. **(C)** Quantification of the slope of input-output plots of EPSP amplitude at SC and TA paths in whole-cell recordings from CA1 pyramidal neurons. A greater slope is proportional to an increased input-output function. Data is shown as cell average from WT (black) and *Fmr1*$^{-/y}$ (red) neurons, with individual neurons shown as open circles. Measurement of paired-pulse ratio (PPR) as derived from a 50 ms inter-pulse interval for all stimulation intensities from EPSP recordings are shown from SC **(D)** and TA **(E)** pathways. **(F)** Quantification of the number of neurons that responded with spiking to any stimulation intensity for WT and Fmr1 neurons, with respect to the stimulation pathway. **(G)** Measurement of the coefficient of variation (CoV) for the onset of action potentials driven by SC or TA afferents. All data is shown as

mean ± SEM, except for (F), where % of neurons is presented. Statistics shown: ns - $p > 0.05$ and * - $p < 0.05$, from 1-way ANOVA (C), GLMM (D, E, G) and Fisher’s exact test (F).

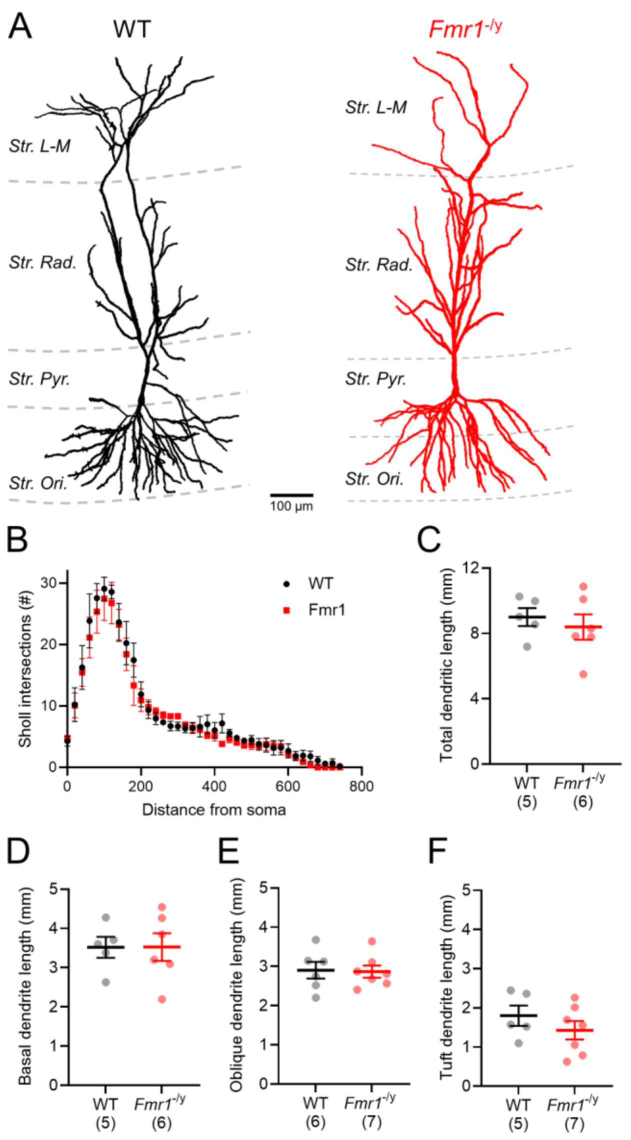

Figure S7. Typical morphology of CA1 pyramidal neurons from *Fmr1$^{-/y}$* rats. (A) 3-dimensional reconstructions of representative CA1 pyramidal neurons from WT (black) and *Fmr1$^{-/y}$* (red) rats, from neurons filled with biocytin during whole-cell patch-clamp recordings. Cells are shown with respect to hippocampal *stratum lacunosum-moleculare* (*Str. L-M*), *Radiatum* (*Str. Rad.*), *Pyramidale* (*Str. Pyr.*), and *Oriens* (*Str. Ori*), which are overlain as grey dashed lines. **(B)** Sholl analysis of reconstructed neurons, plotted as the average for all neurons recorded from WT (black, N=3 rats) and *Fmr1$^{-/y}$* (red, N=4) rats. **(C)** Measurement of total dendritic length of fully reconstructed neurons. Data of each rat are overlaid as filled circles. We observed no differences in the length of basal dendrites **(D)**, apical oblique dendrites **(E)** or apical tuft dendrites **(F)**, as measured between genotypes. Data from additional rats

were included where basal dendrites were not filled sufficiently well for reconstruction. Data is shown as mean ± SEM with statistics from GLMM analysis.

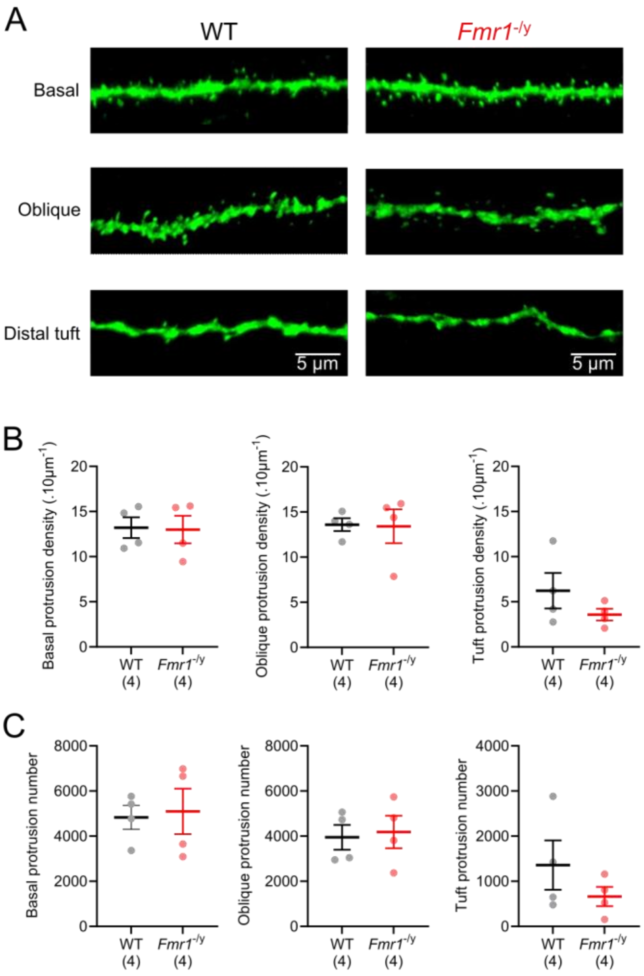

Figure S8. Reduced numbers of apical dendrite protrusions in CA1 pyramidal neurons from the *Fmr*$^{1\text{-}/y}$ rat. (A) Representative deconvolved and flattened confocal z-stacks from basal (upper), apical oblique (middle) and apical tuft (lower) dendrites of biocytin filled CA1 pyramidal neurons, from either wild-type (left) or *Fmr1*$^{-/y}$ (right) rats. (**B**) Quantification of the density of protrusions from the same three dendritic compartments for wild-type (black, N=4) and *Fmr1*$^{-/y}$ (N=4) rats. No difference between genotype was noted for any compartment (basal: $t_{(\text{d.f. }6)}= 0.109$, $p=0.92$; oblique: $t_{(\text{d.f. }6)}= 0.089$, $p=0.93$; apical tuft: $t_{(\text{d.f. }6)}= 1.279$, $p=0.248$; unpaired Student's t-test). **(C)** Arithmetic sum of total protrusion number for the different compartments, based on the reconstructed neurons the dendrites were sampled from. No statistical difference was identified in any compartment, but a tendency towards reduced protrusion number was noted on the apical tuft dendrites (basal: $t_{(\text{d.f. }6)}= 0.233$, $p=0.82$; oblique: $t_{(\text{d.f. }6)}= 0.258$, $p=0.81$; apical tuft: $t_{(\text{d.f. }6)}= 1.186$, $p=0.281$; unpaired Student's t-test). All means are superimposed with the results of individual rats.

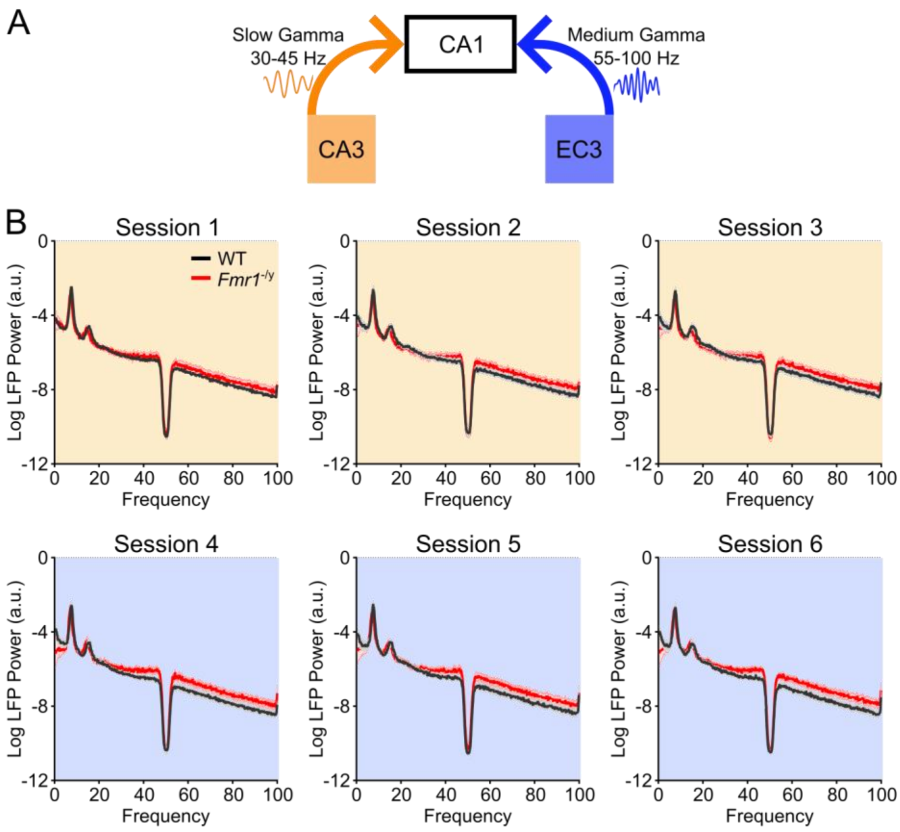

Figure S9. Power of hippocampal oscillatory activity is not significantly different between WT and *Fmr1*$^{-/y}$ rats. (**A**) Schematic depiction of the two major inputs to dorsal CA1. Inputs arriving from CA3 (yellow) are associated with slow gamma neural oscillations. Inputs from MEC3 (blue) are associated with medium gamma neural oscillations. **(B)** Mean LFP power spectra for each session from WT and *Fmr1*$^{-/y}$ rats during the first 4 s of continuous movement following a period of immobility (>3 cm/s). Solid lines depict rat means; shaded areas depict SEM. Pale yellow and pale purple backgrounds denote data from Day 1 and Day 2 respectively.

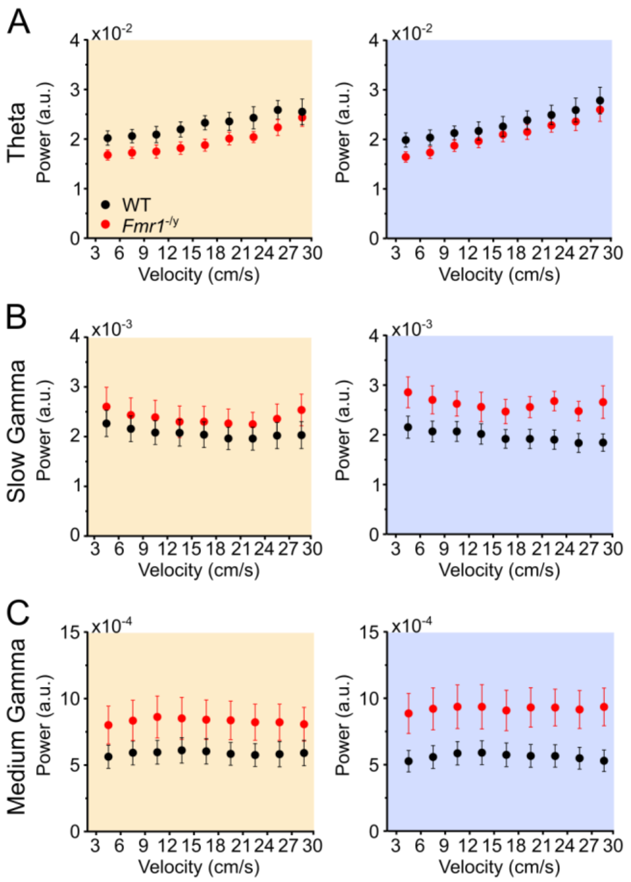

Figure S10. Velocity modulation of hippocampal oscillatory activity power is not significantly different between WT and *Fmr1$^{-/y}$* rats. (A) Theta power plotted as a function of velocity bin. Three-way mixed ANOVA (genotype x day x velocity bin) revealed no significant main effect of genotype and no significant interactions involving genotype, indicating that theta power did not differ as a function of velocity between WT and *Fmr1$^{-/y}$* rats (Genotype: $F_{(1,12)}$=1.474, p=0.248, Day: $F_{(1,12)}$=1.127, p=0.309, Velocity: $F_{(8,96)}$=42.676, p<0.000;. Velocity x genotype x day: $F_{(8,96)}$=1.046 p=0.408; Genotype x day: $F_{(1,12)}$=0.953, p=0.348; Genotype x velocity: $F_{(8,96)}$=1.348, p=0.229; **(B)** Same as (B) for slow gamma power. Again, no significant main effects of genotype or any interactions involving genotype. (Three-way RM ANOVA: Genotype: $F_{(1,12)}$=1.350, p=0.267; Day: $F_{(1,12)}$=1.247, p=0.286; Velocity: $F_{(8,96)}$=3.127, p=0.003; Velocity x genotype x day: $F_{(8,96)}$=0.577 p=0.794; Genotype x day: $F_{(1,12)}$=3.605, p=0.082; Genotype x velocity: $F_{(8,96)}$=1.517, p=0.161.) **(C)** Same as (B) for Medium gamma. No significant main effect of genotype, but the genotype x day interaction was significant. However, *posthoc* testing indicated that medium gamma power did not differ significantly between WT and *Fmr1$^{-/y}$* rats on either Day 1 or Day 2. Rather, *Fmr1$^{-/y}$* (but not WT) medium gamma power differed between Day 1 and Day 2 (Three-way RM ANOVA: Genotype: $F_{(1,12)}$=2.702, p=0.126; Day: $F_{(1,12)}$=1.291, p=0.156; Velocity:

$F_{(8,96)}$=2.472, p=0.018; Velocity x genotype x day: $F_{(8,96)}$=0.919, p=0.524; Genotype x day: $F_{(1,12)}$=6.813, p=0.023; Genotype x velocity: $F_{(8,96)}$=0.833, p=0.576. *Posthoc* tests exploring genotype x day interaction for medium gamma: Two-way ANOVA (genotype x velocity) on Day1: genotype effect $F_{(1,12)}$=1.240, p=0.287; on Day2: genotype effect $F_{(1,12)}$=4.252, p=0.062; Two-way ANOVA (day x velocity) for WT: day effect $F_{(1,6)}$=0.406, p=0.548; *Fmr1*$^{-/y}$: day effect $F_{(1,6)}$= 16.416, p=0.007.) Pale yellow and pale purple backgrounds denote data from Day 1 and Day 2 respectively.

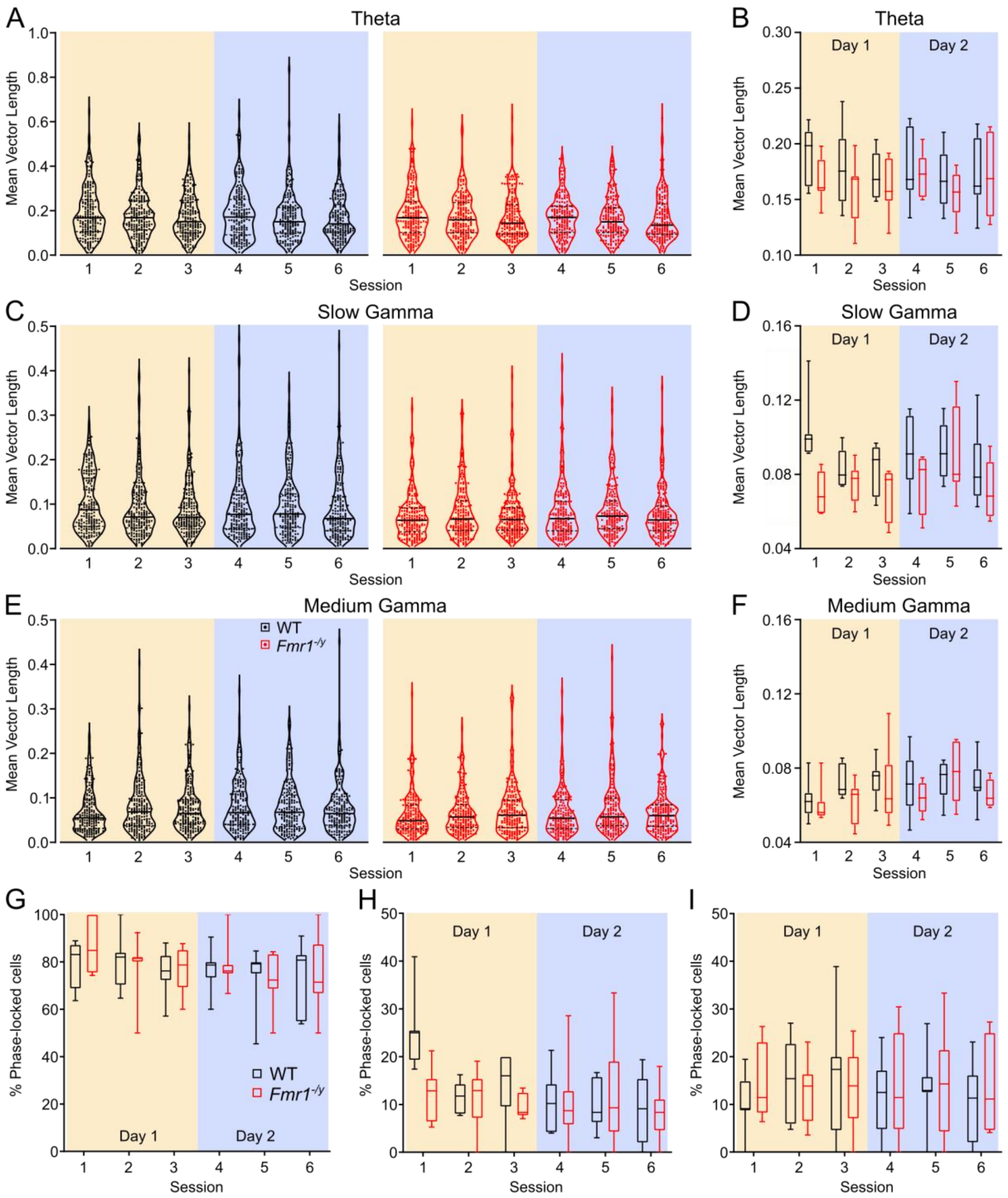

Figure S11. Overview of data distributions for each session and rat means level analysis for spiking modulation by oscillatory activity (A) Violin plots depicting the Theta MVL distributions across all six recording sessions for WT and *Fmr1*$^{-/y}$ pyramidal neurons. **(B)** Theta MVL data plotted and analysed at the rat average level. Three-way RM ANOVA: Day x Session x genotype: $F_{(2,24)}$=0.074, p=0.928; Day x Genotype: $F_{(1,12)}$=0.585, p=0.459; Session x Genotype: $F_{(2,24)}$=0.756, p=0.480; Genotype: $F_{(1,12)}$=0.671, p=0.429; Day: $F_{(1,12)}$=0.372, p=0.553; Session: $F_{(2,24)}$=3.463, p=0.048. **(C-D)** Same as (**A**-**B**) for MVL Slow Gamma. Three-way RM ANOVA: Day x Session x genotype: $F_{(2,24)}$=0.923,

p=0.411; Day x Genotype: $F_{(1,12)}$=1.492, p=0.245; Session x Genotype: $F_{(2,24)}$=4.101, p=0.029; Genotype: $F_{(1,12)}$=5.633, p=0.035; Day: $F_{(1,12)}$=0.637, p=0.440; Session: $F_{(2,24)}$=3.195, p=0.059. *Posthoc* tests Session1&4: WT vs *Fmr1*$^{-/y}$ p=0.005; Session2&5: WT vs *Fmr1*$^{-/y}$ p=0.553; Session3&6: WT vs *Fmr1*$^{-/y}$ p=0.167. **(E-F)** Same as (**A**-**B**) for MVL Medium Gamma. Three-way RM ANOVA: Day x Session x genotype: $F_{(2,24)}$=3.395, p=0.050; Day x Genotype: $F_{(1,12)}$=0.618, p=0.447; Session x Genotype: $F_{(2,24)}$=0.354, p=0.705; Genotype: $F_{(1,12)}$=1.686, p=0.218; Day: $F_{(1,12)}$=3.705, p=0.078; Session: $F_{(2,24)}$=2.798, p=0.081. **(G)** Rat average values of percentages of Theta phase locked pyramidal neurons (Rayleigh p<0.05) across all six recording sessions. Three-way RM ANOVA: Day x Session x genotype: $F_{(2,24)}$=0.025, p=0.976; Day x Genotype: $F_{(1,12)}$=0.004, p=0.951; Session x Genotype: $F_{(2,24)}$=0.014, p=0.986; Genotype: $F_{(1,12)}$=1.830, p=0.201; Day: $F_{(1,12)}$=1.035, p=0.329; Session: $F_{(2,24)}$=0.353, p=0.706. **(H)** Same as (**G**) for Slow Gamma. Three-way RM ANOVA: Day x Session x genotype: $F_{(2,24)}$=2.647, p=0.091; Day x Genotype: $F_{(1,12)}$=2.433, p=0.145; Session x Genotype: $F_{(2,24)}$=3.321, p=0.053; Genotype: $F_{(1,12)}$=1.652, p=0.223; Day: $F_{(1,12)}$=5.376, p=0.039; Session: $F_{(2,24)}$=6.007, p=0.008. **(I)** Same as (**G**) for Medium Gamma. Three-way RM ANOVA: Day x Session x genotype: $F_{(2,24)}$=0.492, p=0.617; Day x Genotype: $F_{(1,12)}$=0.652, p=0.435; Session x Genotype: $F_{(2,24)}$=0.068, p=0.934; Genotype: $F_{(1,12)}$=0.009, p=0.928; Day: $F_{(1,12)}$=0.001, p=0.990; Session: $F_{(2,24)}$=0.660, p=0.526. For box and violin plots the middle line represents rat median, upper and lower end of the boxes, and upper and lower line in the violins represent 95th and 5th percentile, error bars in box plots represent maximum and minimum values. N_{WT} = 7, N_{KO} =7. Pale yellow and pale purple backgrounds denote data from Day 1 and Day 2 respectively.

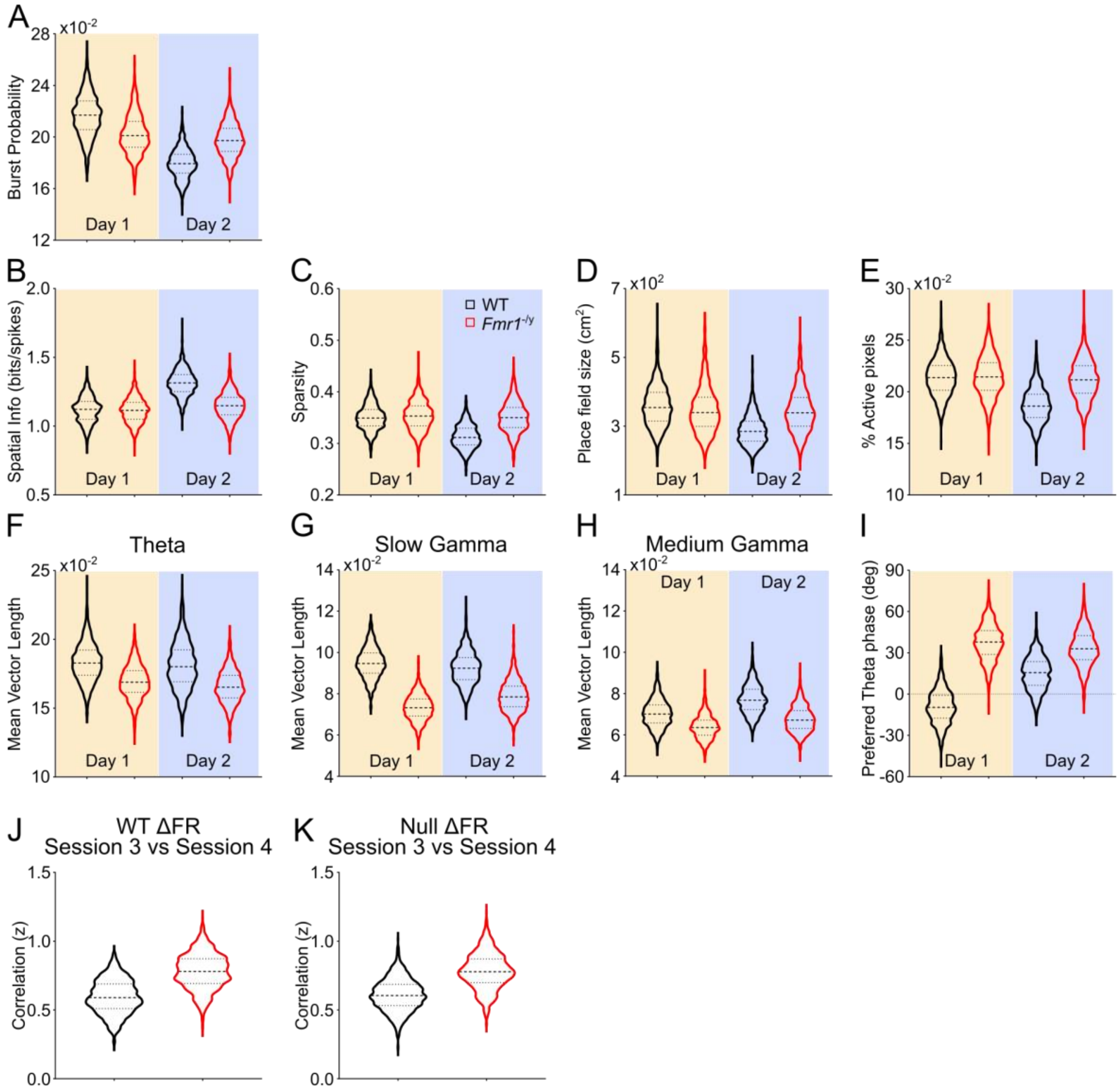

Figure S12. Overview of down sampling analysis examining the possible influence of mean firing rate changes on other measures of cellular activity *in vivo* **(A)** Violin plots depicting the burst probability distributions for WT and *Fmr1*$^{-/y}$ pyramidal neurons across both days of the experiment. Each of the 1000 values in each distribution is the mean value of 30 randomly sampled units. Only when the 30-unit mean was equal to the overall WT-Day2 mean firing rate (+/-5%) was it included in the distribution. (**B**) Same (**A**) for spatial information, (**C**) sparsity, (**D**)Place field size, (**E**) %Active pixels, (**F**) Theta MVL, (**G**) Slow gamma MVL, (**H**) Medium gamma MVL, and (**I**) preferred theta phase. (**K**) Downsampling analysis in which 30 units were randomly selected 1000 times to approximate the WT firing rate change from the last session of day 1 to the first session of day 2. For those cells, the mean correlation between the firing rate map of the two sessions was calculated. (**L**) Same as (**K**) but samples were selected to have no firing rate change between the two sessions (+/- 5% of WT change).
